# Linear Polyethyleneimine-Coated Gold Nanoparticles as a Platform for Central Nervous System Targeting

**DOI:** 10.3390/polym18020298

**Published:** 2026-01-22

**Authors:** Agustín J. Byrne, Antonia Infantes-Molina, Enrique Rodríguez-Castellón, Romina J. Glisoni, María J. Pérez, Patrizia Andreozzi, Barbara Richichi, Marco Marradi, Paula G. Franco, Juan M. Lázaro-Martínez

**Affiliations:** 1 Universidad de Buenos Aires, Facultad de Farmacia y Bioquímica, Buenos Aires 1113, Argentina; abyrne@docente.ffyb.uba.ar (A.J.B.); romy.glisoni@gmail.com (R.J.G.); mjuliaperez@gmail.com (M.J.P.); 2 CONICET—Universidad de Buenos Aires, Instituto de Química y Fisicoquímica Biológicas (IQUIFIB), Buenos Aires 1113, Argentina; 3 Departamento de Química Inorgánica, Cristalografía y Mineralogía, Facultad de Ciencias, Instituto Interuniversitario en Biorrefinerías I3B, Universidad de Málaga, 29010 Málaga, Spain; ainfantes@uma.es (A.I.-M.); castellon@uma.es (E.R.-C.); 4 CONICET—Universidad de Buenos Aires, Instituto de Nanobiotecnología (NANOBIOTEC-UBA-CONICET), Buenos Aires 1113, Argentina; 5 Department of Chemistry ‘Ugo Schiff’, University of Florence, Via della Lastruccia 3-13, 50019 Florence, Italy; patrizia.andreozzi@unifi.it (P.A.); barbara.richichi@unifi.it (B.R.); marco.marradi@unifi.it (M.M.); 6 CONICET—Universidad de Buenos Aires, Instituto de Química y Metabolismo del Fármaco (IQUIMEFA-UBA-CONICET), Buenos Aires 1113, Argentina

**Keywords:** gold nanoparticles, polyethyleneimine, characterization techniques, dark field microscopy, biodistribution

## Abstract

The unique physicochemical properties of gold nanoparticles (GNPs) have made them versatile tools for biomedical applications, such as imaging, therapy, and drug delivery. The surface modification of GNPs with polymers or biomolecules can enhance their colloidal stability and facilitate internalization into cells. However, the efficient and biocompatible delivery to the central nervous system remains a major challenge, as many existing nanocarriers show poor capacity to cross the blood-brain barrier. We developed a method to coat GNPs with linear polyethyleneimine (GNP@PEI) through a chemical reduction bottom-up approach, in which linear PEI hydrochloride acts simultaneously as a reducing and stabilizing agent of colloidal dispersion. This strategy yielded monodisperse spherical GNP@PEI nanoparticles with an average diameter of 50 nm. The physicochemical profile, biocompatibility, and capacity for neural uptake of this potentially brain-targeted nanoplatform were then evaluated. GNP@PEI nanoparticles exhibited high biocompatibility in several primary neural cultures and cell lines, with cellular uptake showing clear cell-type-dependent differences. In vivo studies carried out in a murine model demonstrated that after the intranasal or intraperitoneal administrations of GNP@PEI nanoparticles, detectable levels of gold were found in several organs, including the brain. Collectively, these findings highlight the potential of GNP@PEI as a promising nanoplatform for brain-targeted delivery and for advancing the development of therapeutic strategies for neurological disorders.

## 1. Introduction

Gold nanoparticles (GNPs) have attracted significant interest in biomedical research because of their unique optical, chemical, and structural properties, which support applications in imaging, drug delivery, photothermal therapy, and diagnostics [1,2,3,4,5]. Importantly, the safety and biocompatibility of gold-based nanomaterials have been evaluated in multiple preclinical and clinical studies, supporting their translational potential [6]. A key advantage of GNPs is their highly versatile surface chemistry, which enables functionalization with polymers, biomolecules, or targeting ligands to improve colloidal stability, modulate biological interactions, and enhance cellular uptake [7,8,9,10,11]. However, the nanoparticle behavior in biological systems strongly depends on surface composition, charge, and size, highlighting the need for rational and application-specific design strategies.

Polyethyleneimine (PEI) is a cationic polymer widely used in gene delivery because of its capacity to establish strong electrostatic interactions with negatively charged biomolecules and cellular membranes, as well as its buffering capacity [12,13]. Linear PEI has been reported to exhibit improved biocompatibility, as compared to branched PEI, while maintaining efficient cellular internalization [14]. PEI-coated gold nanoparticles have been shown to display enhanced colloidal stability and cellular uptake [8,15]. The positive surface charge of PEI-coated nanoparticles facilitates membrane association and endocytic internalization through electrostatic interactions [16]. Nevertheless, high surface charge density and inappropriate PEI molecular weight are frequently associated with cytotoxicity, particularly in sensitive tissues such as the central nervous system (CNS). Moreover, despite numerous reports on GNP@PEI systems [15,17,18,19,20,21], there is still poor understanding of how stable PEI-coated gold nanoparticles interact with distinct neural cell populations and whether such systems can achieve effective brain delivery using non-invasive administration routes.

Therapeutic interventions in neurological diseases are markedly limited by the blood–brain barrier (BBB), which prevents systemically administered drugs from entering the brain [22,23,24]. In addition, the CNS represents a highly heterogeneous cellular environment in which neurons, glial cells, and neural stem cells contribute differentially to disease progression, inflammation, and repair. Among them, neural stem cells (NSCs) are multipotent cells capable of generating neurons, astrocytes, and oligodendrocytes and play a central role in brain development and regeneration [25,26]. In addition, microglia, the resident immune cells of the CNS, regulate neuroinflammation, phagocytosis, and tissue remodeling, exhibiting both protective and detrimental roles depending on the inflammatory context [27]. As a consequence, nanocarriers intended for neurological applications must be evaluated not only for general biocompatibility but also for their interactions with distinct CNS cell types that critically influence therapeutic outcomes.

The intranasal administration has emerged as a promising non-invasive strategy to bypass the BBB by enabling direct transport to the brain through the olfactory and trigeminal pathways [28,29]. Although nanoparticle size and surface charge are known to influence the nose-to-brain transport efficiency, systematic studies evaluating PEI-coated gold nanoparticles delivered intranasally—especially regarding their interactions with neural cells and long-term biodistribution—are still limited.

In this study, we report the synthesis and comprehensive characterization of gold nanoparticles coated with 22 kDa linear polyethyleneimine (GNP@PEI) obtained through a one-pot bottom-up approach in which PEI·HCl serves as both a reducing and stabilizing agent. The objectives of this work were to (i) evaluate the physicochemical stability of GNP@PEI, (ii) assess their biocompatibility and cellular uptake in central nervous system–relevant cell types, including neural stem cells and microglia, and (iii) investigate their in vivo biodistribution following intranasal vs. intraperitoneal administration in a murine model. We hypothesized that PEI-stabilized gold nanoparticles synthesized via this strategy can function as a versatile nanocarrier for CNS targeting, combining colloidal stability, favorable interactions with neural cell populations, and the ability to reach the brain through intranasal or intraperitoneal administration. Together, these features position GNP@PEI as a promising platform for the development of nanoparticle-based approaches aimed at neurological, demyelinating, and other central nervous system disorders.

## 2. Materials and Methods

### 2.1. Materials

Hydrogen tetrachloroaurate trihydrate (HAuCl_4_·3H_2_O), pro-analysis nitric acid, poly-L-lysine hydrochloride (P1524), 5-bromo-2′-deoxyuridine (BrdU, catalog #B5002), bisbenzimide H33258 (Höechst 33258, catalog 14530, Frankfurt am Main, Germany), thiazolyl blue tetrazolium bromide (MTT, M2128), and Triton X-100 were purchased from Sigma-Aldrich (St. Louis, MO, USA). Linear polyethyleneimine hydrochloride (PEI·HCl) polymer molecules with a molecular weight of 22, 87, and 217 kDa as free bases were synthesized as described previously [12]. Henceforth, the molecular weights of PEI·HCl samples will be referred to by their corresponding free bases.

Dulbecco′s Modified Eagle′s Medium/Nutrient Mixture F-12 Ham (DMEM-F12) and B27 Supplement were purchased from Gibco Life Technologies, Buenos Aires, Argentina; EGF was purchased from Thermo Fisher Scientific Inc. (Waltham, MA, USA); and bFGF was kindly provided by Dr. Alberto Baldi’s laboratory (IBYME, CONICET, Buenos Aires, Argentina). Plasticware was acquired from Elessar (Buenos Aires, Argentina); fetal calf serum (FCS) from Cripion SRL (Buenos Aires, Argentina); and Mowiol 4-88 Calbiochem (CBC475904) from Research AG (Buenos Aires, Argentina). Sodium pentobarbital was a generous gift from Kangoo Pet Food (Buenos Aires, Argentina). Paraformaldehyde (PFA), DMSO, and other general reagents were from Biopack (Buenos Aires, Argentina).

Primary antibodies anti-Sox2 (ab97959) and anti-Iba-1 (AB5076) were from Abcam Ltd. (Cambridge, MA, USA); anti-PDGFRα (GT15150) was from Neuromics (Edina, MN, USA); anti-β-tubulin III (Tuj1,T8578) was from Sigma-Aldrich (St. Louis, MO, USA); anti-β-tubulin (sc-5274) was from Santa Cruz (Dallas, TX, USA); and anti-BrdU (B2531) was from Roche (Boston, MA, USA). Fluorophore-conjugated secondary antibodies were from Jackson ImmunoResearch Laboratories Inc. (West Grove, PA, USA).

The N2a mouse neuroblastoma cell line (ATCC No. CCL-131), the BV-2 murine microglial cell line [30], the OLN93 rat oligodendroglial cell line [31], and the NIH-3T3 mouse fibroblast cell line (ATCC No. CRL-1658) were kindly provided by Dr. Juana Pasquini’s laboratory (Universidad de Buenos Aires, Buenos Aires, Argentina).

C57-BL6j mice and Wistar rats were maintained at the animal facility at IQUIFIB-UBA-CONICET. All the procedures involving animals were conducted in accordance with the ethical guidelines and approved by the Animal Care and Use Committee of the Facultad de Farmacia y Bioquímica (CICUAL), Universidad de Buenos Aires (REDEC-2024-3374-E-UBA-DCT-FFYB, 9 October 2024; REDEC-2023-926-E-UBA-DCT-FFYB, 24 March 2023; and Exp: 0006360/15, Res: 2429/15, 6 July 2015). Animals were maintained under standard conditions (12-h light/12-h dark cycle, 23 °C, and food and water ad libitum). For the in vivo experiments, the sample size was intentionally limited because the study was conceived as a proof of concept to evaluate GNP@PEI delivery and tissue biodistribution, ensuring compliance with the 3Rs principle by avoiding unnecessary animal use. Animals were randomly divided into different experimental groups: intranasal GNP@PEI administration (30 min, 2 h, 7 d) or intraperitoneal GNP@PEI administration (7 d). Animal welfare was monitored by regular assessment of body weight and daily observation of mobility. No animal exhibited a body weight loss greater than 5% or showed changes in its ability to access food or water; therefore, treatment suspension was not required, in accordance with the criteria established in the protocol approved by the Institutional Animal Care and Use Committee (CICUAL).

#### 2.1.1. GNP@PEI Synthesis

GNP@PEI samples were synthesized through a chemical reduction method using a linear PEI·HCl polymer as both a reducing and stabilizing agent. Glassware was cleaned with freshly prepared aqua regia, thoroughly rinsed with Milli-Q water, and dried before use to prevent contamination with residual metals.

**Scheme 1:**
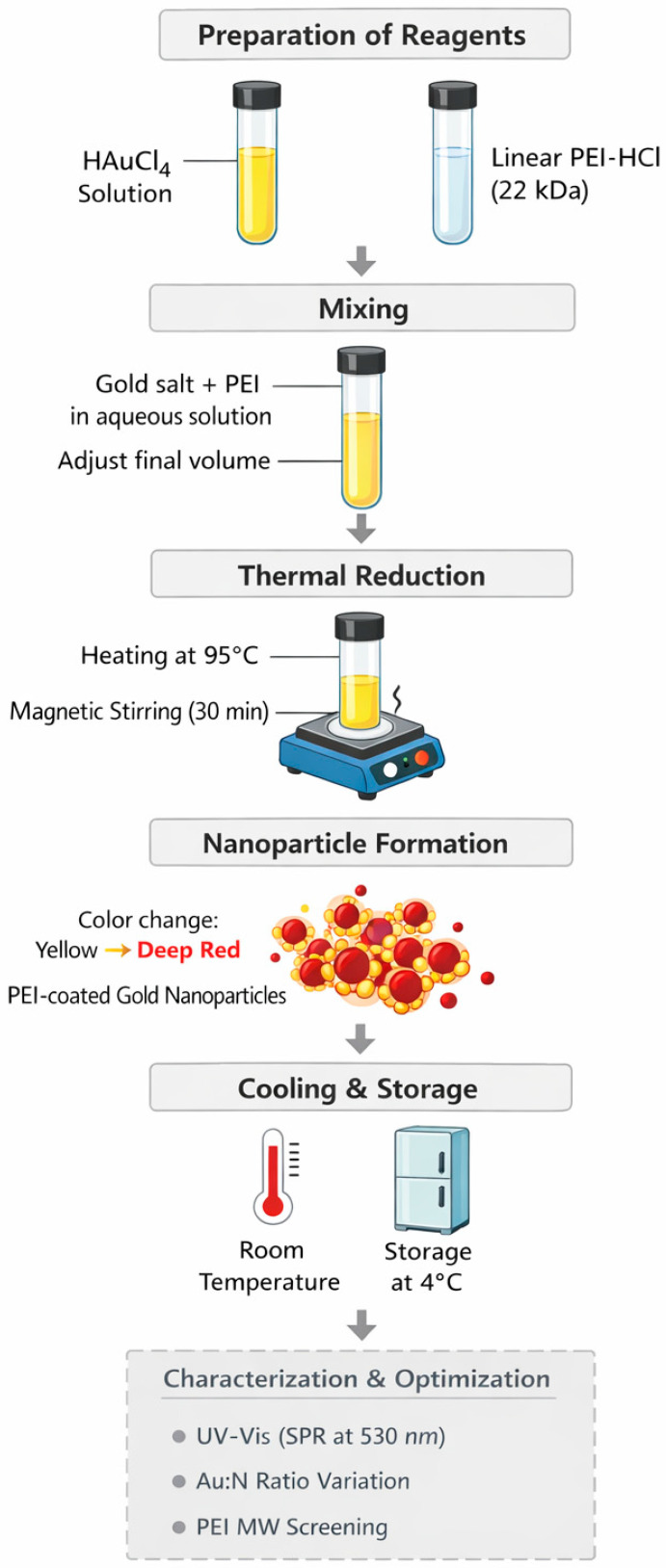
Flowchart for the GNP@PEI synthesis.

For a typical synthesis, 200 μL of an aqueous HAuCl_4_·3H_2_O solution (6 mg/mL) was mixed with 200 μL of linear PEI·HCl (10 mg/mL) in a glass vial, and the final volume was adjusted to 5 mL with Milli-Q water. The reaction mixture was heated at 95 °C under constant magnetic stirring for 30 min. The solution gradually turned deep red, indicating the formation of GNP@PEI. After completion, the reaction was allowed to cool to room temperature and stored at 4 °C for further characterization (Scheme 1). The resulting GNP@PEI dispersion contained approximately 120 μg/mL of gold and 400 μg/mL of PEI·HCl (a total of 520 ppm). The weight percentage (% *w*/*w*) of PEI·HCl and gold in the GNP@PEI material was 77% and 23%, respectively, according to the inductively coupled plasma mass spectrometry (ICP-MS) results.

To evaluate the influence of the polymer molecular weight on nanoparticle formation, linear PEI·HCl samples with different molecular weights were tested under identical conditions. Further optimization was performed using 22 kDa PEI·HCl and varying the gold and nitrogen atoms molar ratio of the polymer (Au:N of 1:3, 1:4, 1:6, 1:13, and 1:26). GNP@PEI formation was confirmed by UV-Vis spectroscopy (Biotek Synergy H1 plate reader, Winooski, VT, USA), showing the characteristic gold surface plasmon resonance (SPR) band at 530 nm. The colloids obtained were stored at 4 °C and remained stable for several weeks without detectable aggregation (Scheme 1).

#### 2.1.2. Synthesis of PEI–RBITC Conjugate for Fluorescent Labeling of GNP@PEI

To generate a fluorescent polymer for nanoparticle synthesis, linear PEI (22 kDa) was covalently modified with rhodamine B isothiocyanate (RBITC). Briefly, 0.9 mL of a 7 mM RBITC solution in DMSO was mixed with 12 mL of PEI·HCl (3.75 µmol) dissolved in 0.02 M NaOH. The reaction mixture was kept under continuous magnetic stirring at room temperature for 72 h to allow covalent binding of RBITC to the secondary amines of PEI. When the reaction was completed, the mixture was transferred to SnakeSkin^®^ Pleated Dialysis Tubing (MWCO = 3500 Da) from Thermo Fisher Scientific Inc. (Waltham, MA, USA) and dialyzed extensively against 2 L of deionized water. After dialysis, the solution was freeze-dried, yielding a purple cotton-like solid, corresponding to the RBITC-labeled PEI polymer (PEI–RBITC). The efficiency of dye conjugation was quantified spectrophotometrically. A standard calibration curve of RBITC (λ_max_ = 555 nm; *R*^2^ = 0.997) was used to determine the amount of dye incorporated in the polymer, resulting in a final ratio of 8 µg RBITC per mg of polymer (PEI:RBITC). This PEI–RBITC conjugate was subsequently employed as the reducing and capping agent in the synthesis of fluorescent GNP@PEI–RBITC, following the same gold reduction protocol used for unlabeled GNP@PEI (see Section 2.1.1).

### 2.2. Characterization Techniques

The hydrodynamic diameter (D*_h_*), polydispersity index (PdI), and zeta potential (ζ-potential) of GNPs were determined by Dynamic Light Scattering (DLS) using a Zetasizer Nano-ZS instrument (Malvern Instruments, Malvern, UK), equipped with a 633 nm He-Ne laser and a digital correlator. Measurements were performed at 25 °C at a fixed backscattering angle of 173° and a laser path length of 4.65 mm. Prior to the analysis, the GNP dispersions were gently homogenized and transferred to disposable cuvettes. The GNP dispersion concentration was 520 ppm (as the GNP@PEI was synthesized) for both size and ζ-potential determinations. Each measurement included at least five runs per sample, and three independent experiments were conducted. Data were expressed as mean ± standard deviation. Analyses were performed using Nano-ZS software (version 7.12, Malvern Instruments).

The GNP synthesis was also done in deuterium oxide (D_2_O) as specified in Section 2.1 and followed by Nuclear Magnetic Resonance (NMR) spectroscopy analysis in a Bruker Avance-III HD spectrometer (Billerica, MA, USA) equipped with a 14.1 T narrow bore magnet operating at a Larmor frequency of 600.09 for ^1^H.

The diamond-attenuated total reflectance Fourier transform infrared (ATR-FTIR) spectrum of GNP@PEI was recorded on a Nicolet iS50 spectrometer (Thermo Fisher Scientific Inc., Waltham, MA, USA) using a one-reflection diamond crystal.

The XPS analysis was carried out with a Physical Electronics Versa-Pro II spectrometer (Chanhassen, MN, USA) operating with a monochromatic X-ray source Al (Ka) of photons at 1486 eV under ultra-high vacuum at a pressure of 10^−6^ Pa. For this technique, the GNP dispersion was frozen and lyophilized to obtain a solid sample.

The high-resolution transmission electron microscopy (HR-TEM) and the scanning transmission electron microscopy (STEM) images were obtained with a TALOS F200X microscope (Thermo Fisher Scientific Inc., Waltham, MA, USA), and the energy dispersive X-ray (EDX) analysis was used to determine the element distribution in the samples. The water GNP@PEI dispersion was dropped onto a perforated copper film prior to the analysis.

### 2.3. Primary Cell Cultures

#### 2.3.1. Primary Neurosphere Cultures from Mouse Brain

Two- to four-day-old newborn mice of both sexes were sacrificed by decapitation, and their brains were removed. For neurosphere cultures, the subventricular zone tissue lining lateral ventricle walls was dissected under a Leica EZ4 stereomicroscope (Deer Park, IL, USA) as a thin 1-mm-thick strip. Cell suspensions were obtained by mechanical dissociation of SVZ tissue with a P1000 micropipette tip. Cells were washed in 5 mL DMEM-F12, and after centrifuging for 5 min at 300× *g*, the supernatant was discarded. Cell pellets were resuspended and cultured for 7–10 d in T-25 flasks with DMEM-F12 supplemented with 2% B-27, 20 ng/mL bFGF, and 20 ng/mL EGF. Growth factors were added to the culture medium every other day.

Neurospheres were allowed to settle by gravity, the culture medium was removed, and the resulting cell pellets were mechanically dissociated. The cell suspension was seeded onto poly-L-lysine-coated coverslips at a density of 20,000 cells per well in a 24-well plate, and after attachment, cells were cultured in the presence of growth factors for at least 24 h prior to treatment. For intact neurosphere analysis, the dissociation step was omitted. Intact or dissociated neurospheres were treated with GNP@PEI for either 24 or 48 h as indicated in Section 3. For viability assays, neural stem cells were seeded at a density of 20,000 cells per well in 96-well plates.

#### 2.3.2. Primary Microglia Culture from Rat Brain

Two-day-old Wistar rats of both sexes were sacrificed by decapitation, and their brains were removed. Meninges were discarded, and a cell suspension was prepared from brain hemispheres by several rounds of mechanical dissociation with a glass Pasteur pipette in DMEM-F12 supplemented with 10% FBS. The cell suspension was seeded in T-75 flasks coated with poly-L-lysine and incubated at 37 °C and 5% CO_2_. The culture medium was replaced every 3 d until cells reached confluence. Microglia cells were detached from other cells in the culture by shaking the flasks at 140 rpm for 1 h. Microglial cells were collected by centrifugation at 300× *g* for 10 min, and cell pellets were resuspended in DMEM-F12 with 10% FBS. Microglia cells were seeded on glass coverslips for immunocytochemical analysis or in 96-well plates for the viability assay at a density of 40,000 cells per well. After 24 h stabilization in culture, microglia were treated with GNP@PEI for 24 h.

#### 2.3.3. Cell Lines Cultures

Cell lines were cultured in DMEM-F12 supplemented with 10% FBS, under standard culture conditions (37 °C and 5% CO_2_ in a humidified incubator). The N2A neuroblastoma cell line, BV-2 microglial cells, and the OLN93 oligodendroglial cell line were seeded at a density of 30,000 cells per glass coverslip coated with poly-L-lysine, whereas NIH3T3 fibroblasts were seeded at the same density on uncoated glass coverslips for immunocytochemical analyses. Cell lines were treated with GNP@PEI for 24 h. For MTT-based viability assays, cell lines were seeded in 96-well plates at a density of 30,000 cells per well.

#### 2.3.4. Cell Treatments

Dilutions of GNP@PEI were prepared in culture medium from a 520 ppm concentrated stock solution. Equal volumes of the different GNP@PEI working solutions were used for cell treatments to reach the final doses of 2, 5, and 10 ppm. For all cell types studied, untreated controls were incubated with the same volume of culture medium without the addition of GNP@PEI.

#### 2.3.5. MTT Assay

Primary cultures or cell lines were treated for 24 h with different doses of GNP@PEI in six replicates. Bright field images of cultures were captured with an Olympus CK inverted microscope (Tokyo, Japan). The MTT assay was performed to quantify mitochondrial metabolic activity, an indirect indicator of cell viability and/or cell proliferation. Cells were seeded in 96-well plates at the densities specified above (20,000–40,000 cells per well, depending on the cell type), allowed to adhere for 24 h, and subsequently treated with GNP@PEI at the indicated concentrations. Cell metabolic activity was assessed using the MTT assay. Briefly, the culture medium was replaced with 100 µL of fresh medium containing 10 µL of a 3-(4,5-dimethylthiazol-2-yl)-2,5-diphenyltetrazolium bromide solution (MTT), and cells were incubated at 37 °C for 1 h to allow the formation of formazan crystals. The culture medium was replaced with 100 µL of DMSO to dissolve the crystals. The spectrophotometric measurements at 550 nm were performed in a BioTek Synergy H1 microplate reader (Winooski, VT, USA). The mean absorbance of controls was considered as 100% of metabolic activity.

### 2.4. Interaction and Cellular Uptake of GNP@PEI

#### 2.4.1. Immunocytochemistry and Dark Field Microscopy

After GNP@PEI treatment (10 ppm), adhered cells were fixed with 4% PFA, washed 3 times in PBS, blocked for 2 h at room temperature in 5% FBS with 0.1% Triton X100, and then subjected to immunocytochemistry for specific markers. Primary antibodies were prepared in 1% FBS, 0.02% Triton X-100 in PBS, and incubated overnight at 4 °C. Rabbit polyclonal anti-Sox2 antibody was used to detect neural stem cells (dilution 1:200); goat polyclonal anti-PDGFRα antibody was used for the detection of oligodendroglial precursor cells (dilution 1:200); mouse monoclonal anti-β-tubulin III (Tuj-1) antibody was used for detection of neuronal cells (dilution 1:200); goat polyclonal anti-Iba-1 antibody was used to detect microglial cells (dilution 1:200); and mouse monoclonal anti-β-tubulin antibody was used to visualize the cell cytoskeleton in fibroblasts (dilution 1:200).

To identify proliferating cells, after a 24 h pulse of BrdU, cells were fixed as previously described and subjected to a protocol of antigen retrieval. Briefly, cells were incubated with 2 N HCl for 20 min at 37 °C, followed by neutralization with 0.1 M sodium borate (pH = 9) for 15 min at 37 °C, and then blocked overnight with 2% FBS. The primary antibody mouse monoclonal anti-BrdU (dilution 1:100) was incubated overnight at 4 °C.

All secondary antibodies (Jackson IR) were diluted 1:1000 in PBS and incubated for 2 h at room temperature. Höechst 33258 was added at 1 μg/mL for nuclei staining. After washing, coverslips were mounted with Mowiol for microscopy analysis.

Dark field and fluorescence images were captured with an Olympus BX50 fluorescence microscope with a DP73 Olympus digital camera and the CellSens Entry 1.8 software.

#### 2.4.2. Confocal Microscopy Analysis of RBITC-Labeled GNP@PEI

RBITC-labeled GNP@PEI was synthesized following the same procedure described in Section 2.1 for the preparation of non-labeled GNP@PEI, using PEI covalently modified with rhodamine B isothiocyanate (RBITC) as the reducing and stabilizing agent (see Section 2.1.2). For the internalization assay, N2a, BV-2, OLN93, and NIH-3T3 cells were seeded at a density of 30,000 cells per coverslip and grown until they reached ~70% confluence. At this point, the serum-containing culture medium was removed, and cells were washed and incubated for 2 h in serum-free medium to induce starvation. Subsequently, cells were incubated with 10 ppm of RBITC-labeled GNP@PEI for 2 additional hours. After incubation, coverslips were thoroughly washed with PBS and fixed in 4% PFA for 10 min. The intracellular localization was confirmed by confocal microscopy using a Zeiss LSM880 confocal laser scanning microscope at the Lanais-Mie core facility at the Instituto de Biología Celular y Neurociencias (IBCN, Buenos Aires, Argentina).

### 2.5. GNP@PEI Administration In Vivo

#### 2.5.1. Intranasal Administration for Nose-to-Brain Delivery of GNP@PEI

Each adult male mouse (4 months old) (*n* = 6) received 2 µL of the GNP@PEI formulation per nostril. The intranasal administration was performed, without anesthesia, while the animal was gently restrained in a supine position, by delivering the droplets alternately into each nostril with a calibrated P2 micropipette. After the intranasal instillation, mice were kept in the same position for approximately 1 min to minimize dripping and ensure nasal deposition. Animals were then maintained under standard conditions until sacrifice at defined timepoints (30 min, 2 h, and 7 d) post-administration. For each time of sampling, two mice were used (*n* = 2). At each timepoint, animals were euthanized by an intraperitoneal injection of sodium pentobarbital (150 mg/kg). A rapid tissue dissection was then performed. Brain regions (olfactory bulbs and cerebral hemispheres) and peripheral organs (liver, kidney, spleen, and lungs) were collected, weighed, and stored at −80 °C until gold quantification by ICP-MS. Given that gold is not an endogenous biological element, all quantified gold corresponds to exogenously administered GNP@PEI. Therefore, no biological control group was included.

#### 2.5.2. Intraperitoneal Administration of GNP@PEI in Adult Mice

For intraperitoneal administration, 4 µL of the GNP@PEI formulation were injected into the intraperitoneal cavity of each adult mouse (*n* = 2) (4 months old). Animals were kept for 7 d under standard conditions (12 light/12 dark cycle, 23 °C, and food and water ad libitum). At the end of the experiment, animals were euthanized by the injection of an intraperitoneal sodium pentobarbital overdose (150 mg/kg), and tissues were removed for gold quantification. Similarly to Section 2.5.1, no biological control group was included.

#### 2.5.3. Gold Quantification

The different samples (GNP@PEI dispersions and tissues) were digested using pro-analysis nitric acid in a MARS-5 microwave digester (CEM Corporation; Matthews, NC, USA) (power = 400 W; pressure (max) = 350 psi; temperature (max) = 165 °C; time = 15 min). The acid employed in the digestion was previously ultrapurified (sub-boiled) using a Berghof Distillacid BSB-939-IR distiller (Eningen, Germany). The total gold content determination was performed in an inductively coupled plasma spectrometer with atomic mass detection, equipped with a high-purity helium crossflow collision cell (Shimadzu 2030 ICP-MS-CC, Kyoto, Japan). In all cases, Chem-Lab NV certified standards (Zedelgem, Belgium) were used for external calibration. Gold detection in tissue samples was performed by an operator blinded to the experimental groups.

#### 2.5.4. Statistical Analysis

The statistical analysis was performed with the GraphPad Prism software version 10.6.1 (Boston, MA, USA) (RRID:SCR_002798). Statistical tests are indicated in figure legends, and significance is represented as *** *p* < 0.001, ** *p* < 0.01, * *p* < 0.05. Bars without asterisks or indicated as ns correspond to non-significant differences.

## 3. Results

### 3.1. Synthesis and Characterization of PEI-Gold Nanoparticles (GNP@PEI)

The synthetic strategy for preparing the GNPs was based on a previous report [21] in which the 25 kDa linear PEI free base polymer was either dissolved in an acidic medium or heated to favor its dissolution in water. Herein, the linear PEI was used as hydrochloride (PEI·HCl) because this form is highly soluble in water and allows batch-to-batch reproducibility. In this sense, linear PEI·HCl polymers have been investigated as both a reducing and stabilizing agent for gold nanoparticle synthesis. This strategy also offers a greener alternative to traditional chemical reducers, such as sodium borohydride, affording an eco-friendly approach aligned with green chemistry principles [32]. PEI·HCl polymers were selected on the basis of their previously reported low toxicity on bovine fetal fibroblast primary cultures [13] as well as prior evidence from our group demonstrating their effectiveness as GNP stabilizers by electrodeposition on glassy carbon electrodes [33].

First, the PEI·HCl polymers with different molecular weights were analyzed to evaluate their cytotoxic effect in neural stem cell primary cultures. Proliferating neural stem cells were treated with linear PEI·HCl of different molecular weights for 24 h, and the cell metabolic activity was determined by the MTT assay. Our results showed a dose-dependent reduction in the metabolic activity with all the tested polymers (Figure S1). Of all the different polymer molecular weights, the 22 kDa PEI·HCl exhibited the lowest cytotoxic effect, as evidenced by the ≥60% cell viability obtained with the highest tested concentration (5 μg/mL). The differences obtained with the different polymers were consistent with the expected variation in the physicochemical behavior related to the polymer molecular weight. These results were in agreement with previous ones obtained with PEI·HCl in bovine fetal fibroblast primary cultures [13].

**Figure 1:**
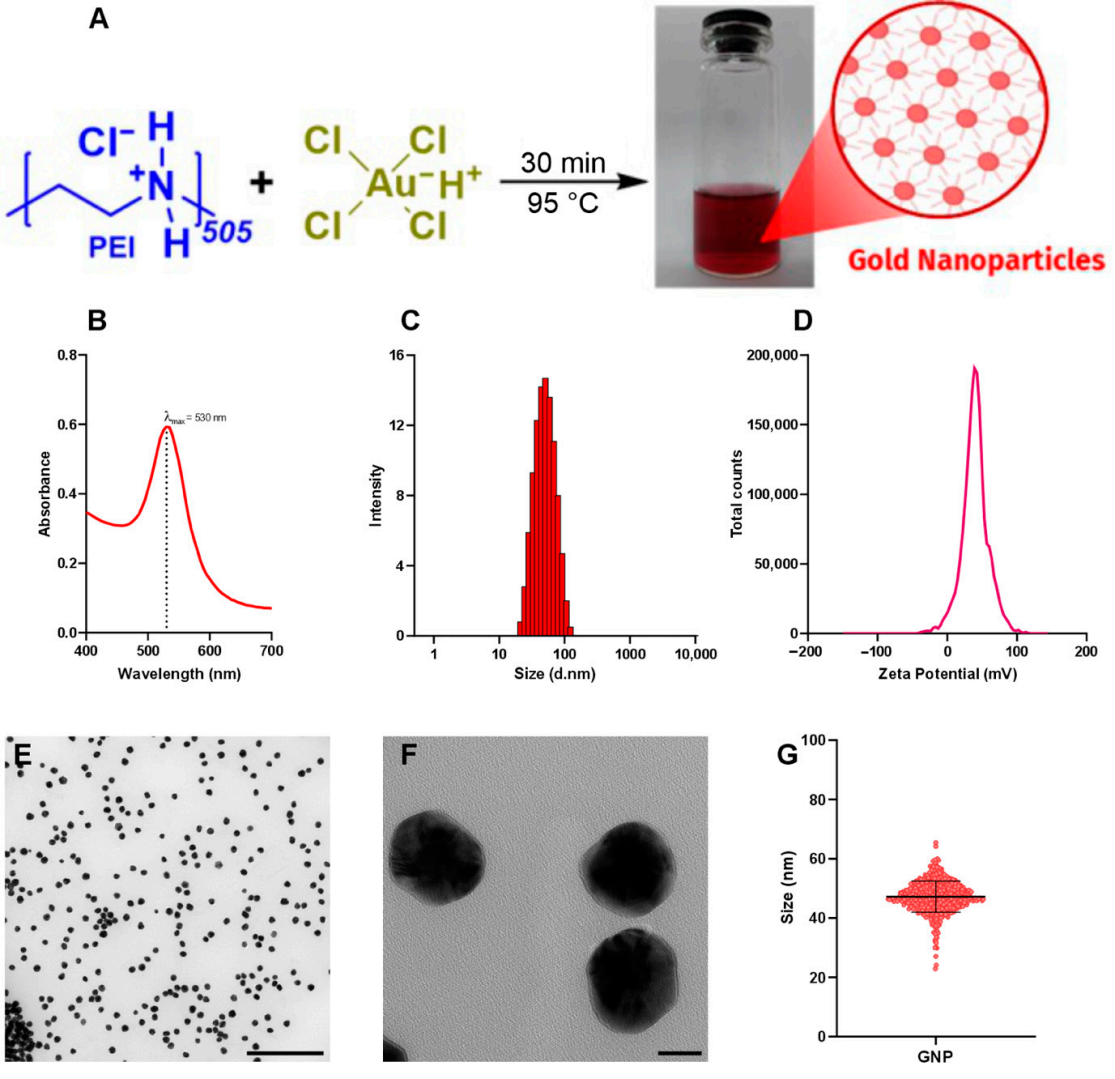
Synthesis and structural characterization of PEI-stabilized gold nanoparticles (GNP@PEI). (**A**) Schematic illustration of the synthesis of GNP@PEI obtained by reacting linear PEI·HCl (22 kDa) with HAuCl_4_ for 30 min at 95 °C. (**B**) UV–Vis absorption spectrum of GNP@PEI showing the surface plasmon resonance (SPR) band with a maximum at λ = 530 nm. (**C**) Size distribution by intensity (%) and (**D**) ζ-potential distribution for GNP@PEI measured by dynamic light scattering (DLS) in deionized water at 25 °C. (**E**,**F**) Representative HR-TEM micrographs of GNP@PEI at different magnifications. (**G**) Particle size distribution determined from HR-TEM images (*n* ≈ 600 particles), yielding an average diameter of 47 nm. Scale bars in (**E**,**F**) represent 500 and 20 nm, respectively.

GNP@PEI was synthesized through a chemical reduction bottom-up method (Figure 1A). The one-step synthesis carried out under heating enables the reduction of Au^3+^ ions from HAuCl_4_ by PEI amino groups, yielding well-dispersed polymer-stabilized GNPs within 30 min. The characteristic color transition from yellow to red, which confirms the formation of colloidal gold (Video S1), occurred with high temporal reproducibility across independent batches.

To evaluate the influence of the polymer molecular weight on nanoparticle formation, GNP@PEI was synthesized using linear PEI·HCl polymers of 22, 87, and 217 kDa with an Au:N molar ratio of 1:6, according to a previously reported study [21] and defined as the proportion between gold atoms and PEI nitrogen groups. UV-Vis spectra of all formulations exhibited similar surface plasmon resonance bands, whereas the DLS analysis showed that GNP@PEI synthesized with 22 kDa PEI·HCl displayed the smallest hydrodynamic diameter (D*_h_*), the lowest polydispersity index (PdI), and high zeta potential, which favors colloidal stability (Figure S2A–D). Based on these findings and considering that 22 kDa PEI·HCl showed the lowest cytotoxicity in neural stem cell cultures, this polymer was selected for subsequent GNP syntheses.

Then, different Au:N molar ratios were assessed to analyze which reaction conditions enabled the generation of a homogeneous and stable GNP@PEI using the 22 kDa PEI·HCl polymer. At an Au:N molar ratio of 1:6, the smallest hydrodynamic diameter and PdI and the most homogeneous population were obtained, as evaluated by DLS (Figure S2E,F). Therefore, this Au:N ratio was chosen for further optimization and characterization experiments. UV–Vis spectra showed the presence of the characteristic surface plasmon resonance band between 530 and 540 nm for all ratios. The 1:6 Au:N ratio allowed the production of a monodisperse and stable colloidal suspension, ensuring reproducible results with a narrow surface plasmon band at 530 nm (Figure 1B). DLS studies provided detailed insights into the hydrodynamic behavior of the synthesized nanoparticles in aqueous medium, revealing an average D*_h_* of 53 ± 1 nm (Figure 1C), with a narrow size distribution (PdI = 0.169 ± 0.006). These D*_h_* values corresponded to the gold core plus the hydrated polymeric corona. The intensity distribution displayed a single, dominant nanoparticle population (100%), supporting the formation of a homogeneous GNP@PEI dispersion. The zeta potential of +40 mV (Figure 1D) indicated that the colloidal system was highly stable, as values above +30 mV reflect strong electrostatic repulsion between particles, preventing aggregation [32,34]. HR-TEM analyses were performed to further characterize the morphology and size distribution of GNP@PEI (Figure 1E,F). The micrographs revealed uniform, spherical nanoparticles with an average core diameter of approximately 47 nm (Figure 1G), in agreement with the nanometric dimensions estimated by DLS (53 ± 1 nm). These structural parameters were those of the nanoparticles used in the subsequent cellular assays.

To evaluate the long-term colloidal stability of the GNP@PEI suspension, DLS measurements were periodically performed over a six-month period. Both the hydrodynamic diameter and PDI values remained unchanged, indicating that the nanoparticles preserved their size and dispersity without any evidence of aggregation (Table 1, Figure S3). This remarkable stability can be attributed to the strong electrostatic repulsion between particles conferred by the highly positive surface charge of protonated PEI chains surrounding the gold cores. No additional pH adjustment was required during the synthesis, and pH values of the dispersion remained within the expected range for PEI·HCl solutions.

Although GNP@PEI colloids remained stable at 4 °C for several months, as confirmed by DLS measurements, lyophilization was tested as an alternative long-term storage strategy. This process disrupted the colloidal suspension, leading to extensive nanoparticle aggregation and growth, as indicated by the SPR band broadening observed in the UV-Vis spectrum (Figure S4A) and the increased particle size (Figure S4B) observed by HR-TEM (Figure S4C–F). HAADF-STEM imaging combined with EDX elemental mapping for Au and N confirmed the distribution of gold surrounded by nitrogen-containing polymeric material (Figure S4G–L). These results indicate that the lyophilization protocol used did not preserve colloid stability. Optimization of the drying procedure is necessary to prevent aggregation.

Further analyses by EDX revealed that the spherical particles presented a homogeneous distribution of all the elements that compose the GNP@PEI, with a higher proportion of gold (80 wt%) as compared to the nitrogen (5 wt%) and chlorine content (15 wt%) (Figure S5). In addition, the chemical surface composition of the GNP@PEI systems was assessed by XPS. The atomic concentration (at%) for carbon (C 1*s*), nitrogen (N 1*s*), oxygen (O 1*s*), chloride (Cl 2*p*), and gold (Au 4*f*), determined in the GNP@PEI by XPS, was 61.8 (47.2 wt%), 16 (14.2 wt%), 11 (11.2 wt%), 11 (24.9 wt%), and 0.2 (2.5 wt%), respectively. In particular, the contribution of oxygen in the GNP@PEI was attributed to the inclusion of water molecules during the lyophilization process of the nanoparticle dispersion for XPS analysis. The high-resolution core level Au 4*f* spectrum of the GNP@PEI shows binding energies of Au 4*f*_5/2_ and Au 4*f*_7/2_ at ≈87.5 and ≈83.8 eV, respectively (Figure S6 and Table S1), which are typical values of metallic Au (Au^0^), confirming that the HAuCl_4_ was completely reduced by PEI·HCl [35].

**Figure 2:**
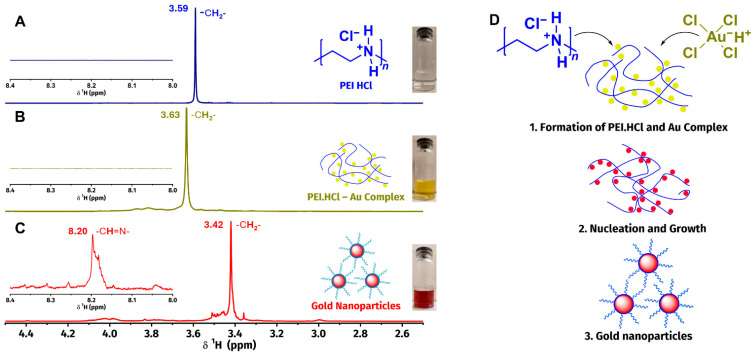
Structural analysis and proposed formation mechanism of GNP@PEI. ^1^H-NMR spectra in D_2_O of (**A**) linear PEI·HCl (22 kDa), (**B**) PEI-Au complex, and (**C**) GNP@PEI. The characteristic methylene peaks of PEI are visible in all samples, while a new signal appears at 8.20 ppm in (**C**), attributed to imine (-CH=N-) formation upon PEI oxidation during nanoparticle synthesis. (**D**) Schematic representation of the proposed steps in GNP@PEI generation: (1) PEI-Au complexation, (2) nucleation and growth, and (3) final stabilization of GNP@PEI.

To monitor the chemical changes during the synthesis of GNP@PEI, the reaction was performed in D_2_O and analyzed by ^1^H-NMR. PEI·HCl is known to exchange chloride ions for tetrachloroaurate ions (AuCl_4_^−^) prior to Au^3+^ reduction to metallic Au^0^ [33]. In our spectra, the ethylene protons of PEI generated a well-defined resonance peak at δ = 3.59 ppm (Figure 2A), shifting slightly to 3.63 ppm upon addition of HAuCl_4_, along with the appearance of new signals at 3.70–3.90 ppm (Figure 2B). Heating the PEI·HCl/HAuCl_4_ mixture at 95 °C for 30 min induced oxidation of the secondary amino groups (-CH_2_-NH-) to the corresponding imines, which was evidenced by the appearance of a new signal at 8.20 ppm (-CH=N-), concurrent with the reduction of Au^3+^ to Au^0^ and the corresponding formation of the PEI-stabilized GNPs (Figure 2C). Additional resonance signals around δ ≈ 4 ppm indicated interactions of other PEI moieties with the nanoparticle core, while the bulk PEI signal appeared at 3.42 ppm post-synthesis. Overall, these results suggest that the GNP@PEI formation proceeds through three sequential steps (Figure 2D), as previously proposed for branched PEI-coated gold nanoparticles [19,20].

Particularly, the imine formation cannot be simply inferred from the high-resolution C 1*s* and N 1*s* core level spectra due to the possible overlapping with the carbamate carbon (≈287.6 eV) and the protonated nitrogen signals (≈401.2 eV), respectively (Figure S6). The carbamate group is typically found in the ^13^C-NMR spectra at around 163 ppm as a result of the chemical reaction of PEI materials with carbon dioxide in solution [36]. In this context, the ^1^H-NMR results provided bulk information of the nanosystem, allowing the visualization of the imine moieties in the GNP@PEI (Figure 2).

Interestingly, the ATR FT-IR spectrum of the GNP@PEI material indicated the presence of the protonated PEI, as evidenced by the characteristic strong intermolecular hydrogen bonding network in the range of 2900–2300 cm^−1^ [37]. Some changes in the stretching modes were observed when compared with the PEI·HCl material, probably due to the interaction with gold nanoparticles. The strong stretching band of the C=N bond of the imine moieties was detected at 1646 cm^−1^, providing further evidence of the oxidation of amine groups during the concomitant reduction and nucleation of Au^3+^ to Au^0^ (Figure S7).

### 3.2. Effects of GNP@PEI on Cell Viability in Primary Cultures

The effects of GNP@PEI on cell metabolic activity were examined in primary cultures of neural stem cells and microglia, two brain cell types that have emerged as promising targets for therapeutic strategies for neurological diseases.

**Figure 3:**
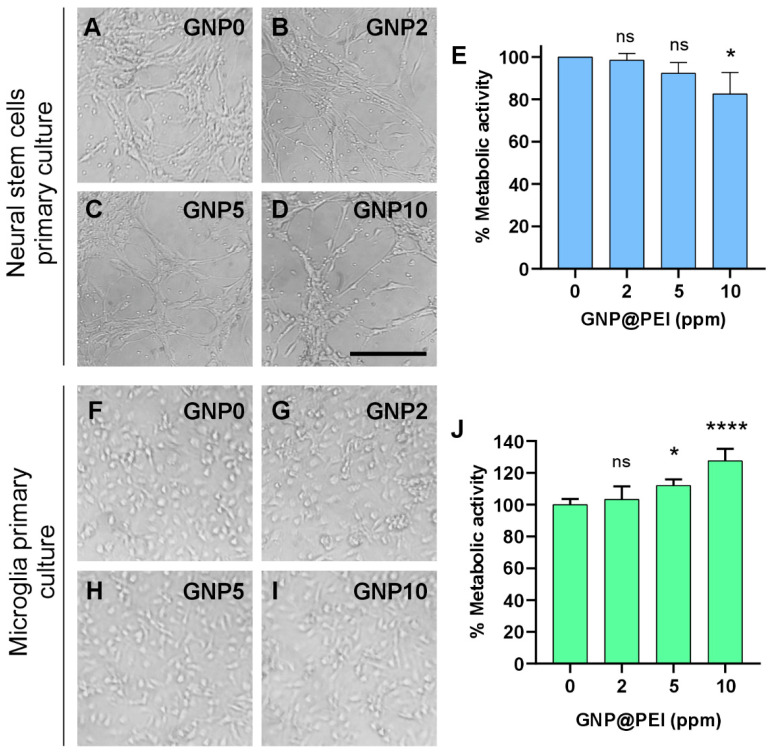
Effects of GNP@PEI treatment on the metabolic activity of primary cultures. (**A**–**D**) Bright field images of neural stem cell primary cultures treated with GNP@PEI at different doses (0, 2, 5, and 10 ppm). (**E**) Mitochondrial activity of neural stem cell cultures assessed by the MTT assay (untreated cells were considered 100%). (**F**–**I**) Bright field images of microglia cell primary cultures under GNP@PEI treatments. (**J**) Metabolic activity of microglia cell cultures assessed by the MTT assay (untreated cells were considered 100%). Panels (**E**,**J**) show the mean values from independent experiments (*n* = 3 for NSCs and *n* = 4 for microglia). Bars represent the mean ± standard deviation (SD). In each experiment, all conditions were performed in six replicates. Asterisks indicate significance vs. controls: * *p* < 0.0332, **** *p* < 0.0001 (one-way ANOVA and Dunnet’s multiple comparison test). The scale bar in (**D**) represents 100 µm for (**A**–**D**,**F**–**I**).

Bright-field microscopy studies showed that the morphology of GNP@PEI-treated neural stem cells was not significantly affected in comparison to controls (GNP0), although a reduction in cell density was observed in treated cells versus controls at higher doses of nanoparticles (Figure 3A–D). The MTT assay revealed that mitochondrial activity was significantly affected by GNP@PEI only at 10 ppm (Figure 3E).

The exposure of primary microglial cell cultures to GNP@PEI did not affect cell viability, as assessed by the MTT assay (Figure 3J). Interestingly, a dose-dependent increase in metabolic activity was observed at higher GNP@PEI concentrations, suggesting enhanced mitochondrial function, cellular activation, or a higher proliferation rate of microglia.

### 3.3. Detection and Visualization of GNP@PEI in Primary Cell Cultures

**Figure 4:**
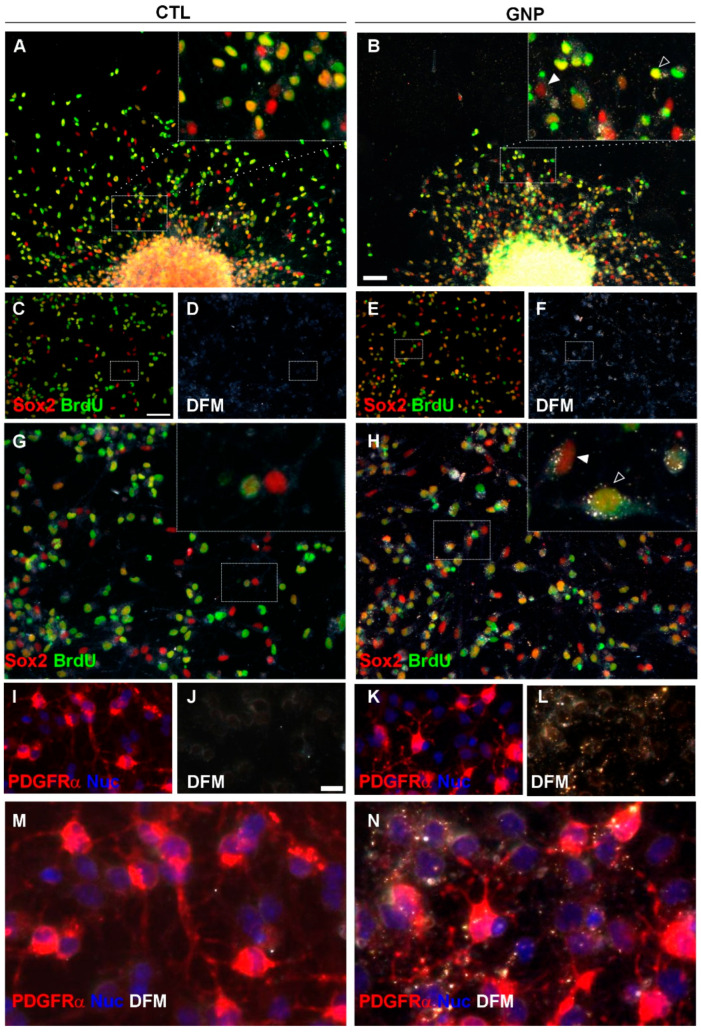
Detection of GNP@PEI particles in neural stem cell cultures by dark field microscopy. Cultures of intact (**A**,**B**) or dissociated (**C**–**H**) neurospheres were analyzed by immunofluorescence. The detection of nanoparticles was done by dark field microscopy (DFM) after 48 h of GNP@PEI or control treatment. (**A**–**H**) The immunofluorescence for Sox2 neural stem cell marker (red) was combined with the detection of BrdU^+^ proliferating cells (green). (**I**–**N**) Oligodendroglial precursor cells were identified by the expression of PDGFR⍺. Nanoparticles were visualized by DFM, and nuclei were stained with Höechst 33258 (blue). Arrowheads and open arrowheads in (**B**,**H**) insets mark non-proliferating (BrdU^−^) and proliferating (BrdU^+^) cells, respectively. The scale bar in (**A**,**C**) represents 50 µm, and the scale bar in (**J**) represents 15 µm.

For imaging of nanoparticles, neural stem cells were grown as neurospheres and were subsequently adhered to polylysine-coated coverslips. After 24 h, adhered neurospheres were treated with GNP@PEI for 48 h in fresh medium containing growth factors. For the identification of proliferating cells, a pulse of BrdU was performed during the last 24 h of treatment. It is well known that under proliferative conditions, neurosphere cells expand the core structure, while numerous cellular processes extend outward to guide progenitor migration (Figure 4A,B) [38]. The intrinsic optical properties of GNPs enable their direct visualization in cell cultures using dark-field microscopy (DFM). In untreated control cultures, cells exhibited the typical pale white contour and diffuse cytoplasmic signal (inset in Figure 4A). In contrast, GNP@PEI-treated cultures displayed numerous bright yellowish, highly refractive spots characteristic of GNPs, detectable both within the neurosphere core and in migrating cells (Figure 4B, inset). These refractive spots showed a heterogeneous distribution among cells in the culture; nevertheless, GNP@PEI was detected in both proliferating Sox2^+^/BrdU^+^ cells (open arrowhead in Figure 4B, inset) and non-proliferating Sox2^+^/BrdU^−^ cells (arrowhead in Figure 4B, inset).

A further analysis of the GNP@PEI-cell interaction was performed in adherent neurosphere-dissociated cells (Figure 4C–H). Merged immunofluorescence and dark-field microscopy (DFM) images revealed the presence of nanoparticles in both proliferative Sox2^+^/BrdU^+^ neural stem cells (open arrowhead in Figure 4H, inset) and non-proliferative Sox2^+^/BrdU^−^ cells (arrowhead in Figure 4H, inset). GNP@PEI was also detected in other proliferative precursors (Sox2^−^/BrdU^+^). The immunostaining for PDGFRα, which in combination with Sox2 labels most of the cells in proliferating neurosphere cultures, revealed the interaction of nanoparticles with oligodendroglial precursor cells (Figure 4I–N). Although DFM revealed the presence of widespread bright refractive spots compatible with GNP@PEI in the culture, this technique does not allow distinguishing whether nanoparticles are internalized or remain associated with the cell surface, or both.

**Figure 5:**
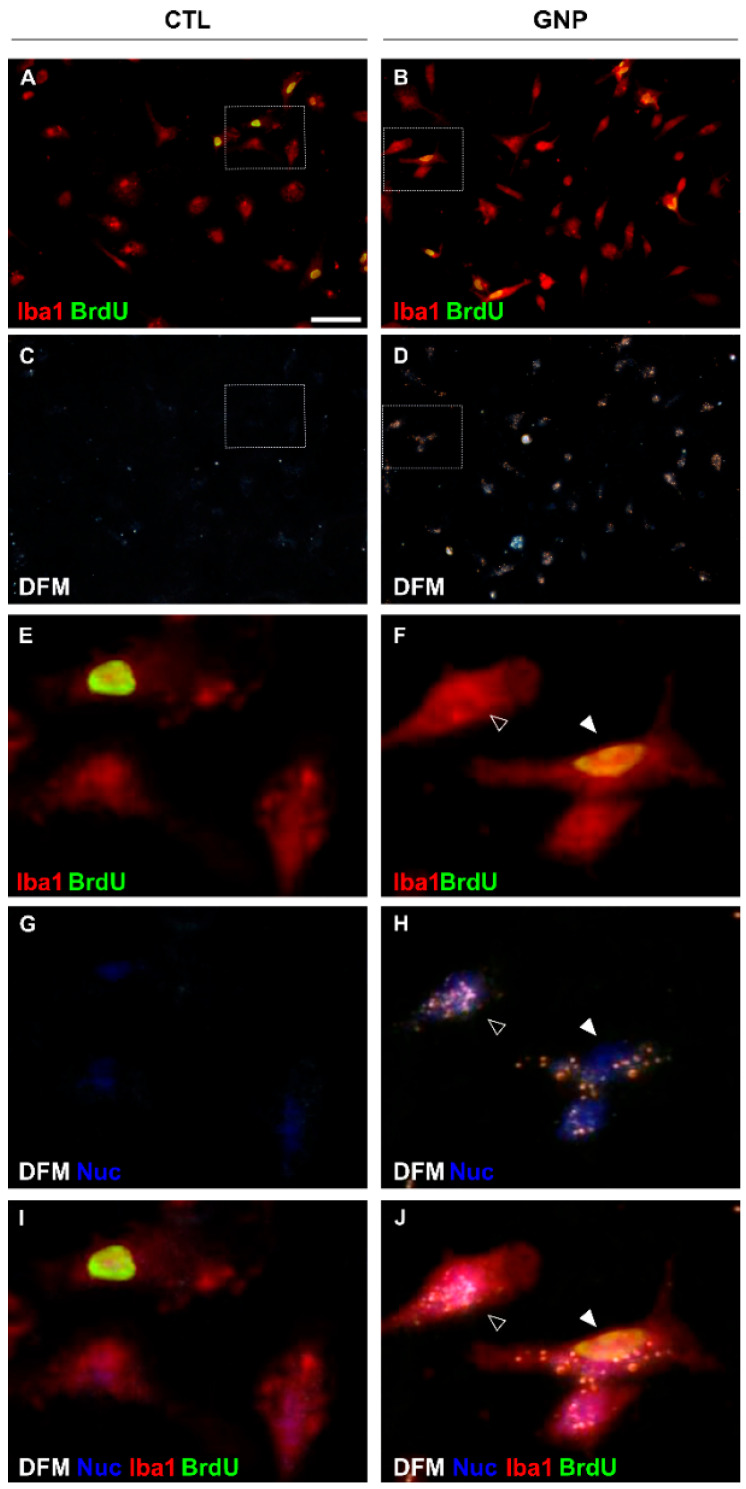
Detection of GNP@PEI in primary microglia cell culture by dark field microscopy. Representative images of cultures without (**A**,**C**) or with GNP@PEI treatment (**B**,**D**). The microglia marker Iba-1 (red) was combined with the detection of BrdU^+^ proliferating cells (green) in (**A**,**B**). (**C**,**D**) Dark field images of cultures. (**E**–**J**) Higher magnification images corresponding to the marked areas in (**A**–**D**), showing overlapping images for the different markers. Arrowheads and open arrowheads in (**F**,**H**,**J**) mark proliferating (BrdU^+^) and non-proliferating (BrdU^−^) cells, respectively. Nuclei were stained by Höechst 33258 (blue). The scale bar in (**A**) represents 50 µm.

Additionally, similar treatments with GNP@PEI were performed in microglia cell primary cultures to evaluate the nanoparticle interaction with cells by dark field microscopy (Figure 5A–J).

Treated cultures were analyzed by BrdU incorporation and Iba-1 immunofluorescence (Figure 5A,B) in combination with DFM (Figure 5C,D). Image insets (Figure 5E–J) revealed the presence of nanoparticles in GNP@PEI-treated microglial cells. The nanoparticles were associated with both proliferating (BrdU^+^) and non-proliferating (BrdU^−^) microglia (Figure 5F,H,J). Given the phagocytic nature of microglia, the dynamics of GNP@PEI uptake was further analyzed by dark-field time-lapse microscopy (Video S2). The nanoparticle internalization was evident immediately after exposure; i.e., within seconds, small vesicle-like structures containing bright spots were seen entering the cytoplasm, indicating rapid endocytosis.

### 3.4. GNP@PEI Treatments Do Not Affect Cell Viability in Neural and Non-Neural Cell Lines

To evaluate the cytotoxic effects of GNP@PEI on other cell types, several cell lines were incubated with increasing concentrations of nanoparticles, assessing cell viability by the MTT assay. Our analysis was focused on brain cell lines, such as OLN93 (oligodendroglial lineage), N2A (neuronal lineage), and BV2 (microglial cells), and on the non-neural cell line NIH3T3 (fibroblast cell line).

**Figure 6:**
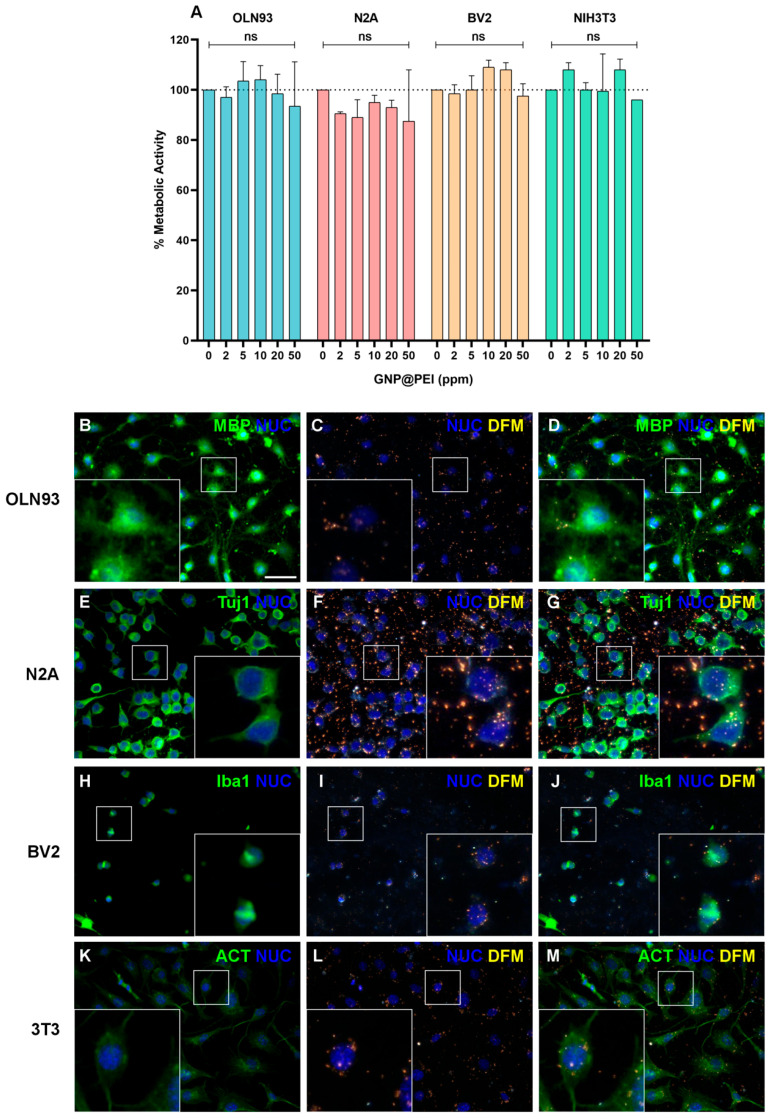
GNP@PEI treatment does not affect cell viability in neural and non-neural cell lines. (**A**) MTT assay after GNP@PEI treatment in OLN93, N2A, BV-2, and NHI-3T3 cells. Bars represent the mean and standard deviation (SD) from two independent experiments (*n* = 2). We performed six replicates for each condition. Data was normalized to controls. The statistical analysis was performed by one-way ANOVA and the Dunnett’s post-hoc test. Differences between GNP@PEI doses and controls for each cell line were not significant (ns). (**B**–**M**) Immunofluorescence images for PDGFRα (**B**,**D**), Tuj1 (**E**,**G**), Iba1 (**H**,**J**), or ꞵtub (**K**,**M**) were combined with DFM detection of nanoparticles (**C**,**D**,**F**,**G**,**I**,**J**,**L**,**M**). Nuclei were stained by Höechst 33258 (blue). The scale bar in (**B**) represents 50 µm.

No significant differences in the levels of metabolic activity were observed between GNP@PEI-treated cells and untreated controls in all tested cell lines (Figure 6A), suggesting that the nanoparticles did not affect cell viability.

In agreement with previous results obtained in primary cell cultures, the DFM analysis revealed the presence of gold nanoparticles as bright yellowish puncta in all analyzed cell lines after GNP@PEI treatment (Figure 6B–M), indicating the interaction of nanoparticles with cells. As a comparative approach, a semi-quantitative analysis of intracellular GNP-associated bright reflective puncta was performed in the different cell lines analyzed. The number of yellow, highly reflective puncta per cell was manually quantified from dark-field microscopy images. This analysis revealed cell-type-dependent differences in the extent of intracellular GNP-associated signal. OLN93 cells displayed an average of approximately 20 ± 8 puncta per cell, whereas N2A cells showed a lower number, averaging 11 ± 5 puncta per cell. BV-2 microglial cells exhibited an intermediate level of intracellular puncta (17 ± 7 per cell), while 3T3 fibroblasts showed the highest number, with 25 ± 8 puncta per cell.

### 3.5. Studies on GNP@PEI Internalization into Cells

**Figure 7:**
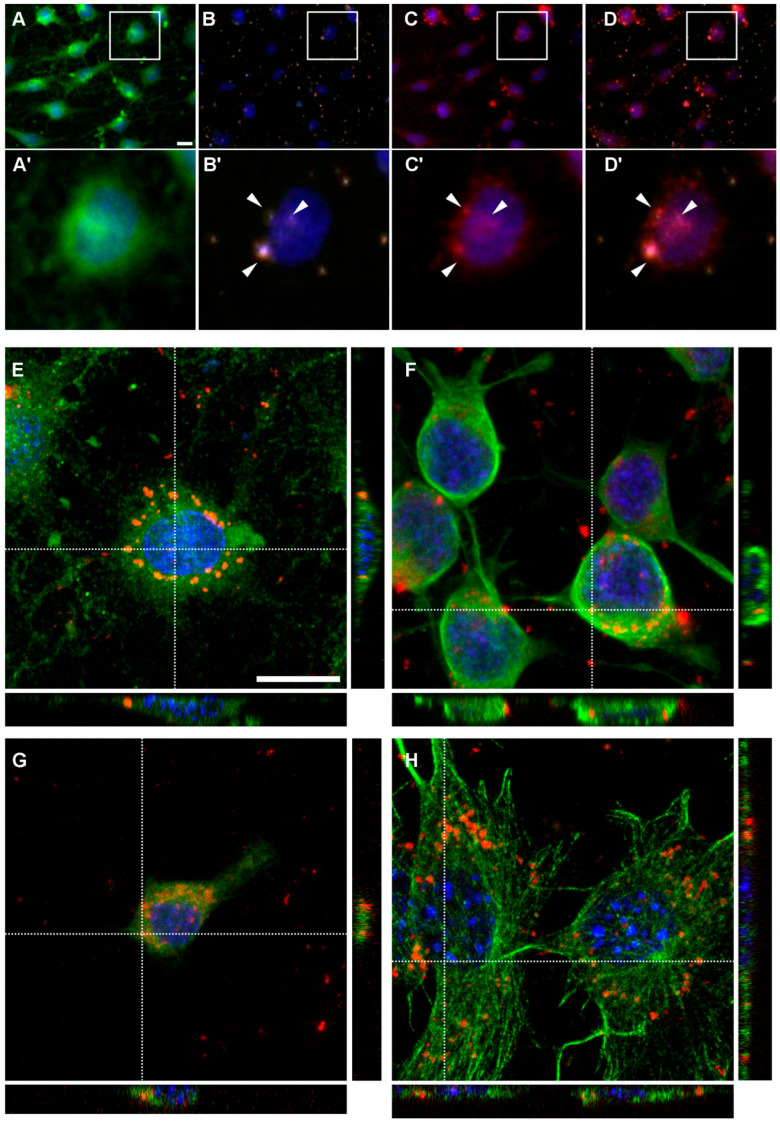
Studies on RBITC-labeled GNP@PEI uptake by different cell types. (**A**–**H**) Uptake and intracellular distribution of GNP@PEI-RBITC (red) in different cell types. Höechst-stained nuclei are in blue. (**A**–**D**) Immunofluorescence for PDGFRα (green) and dark field microscopy (DFM) of OLN-93 cultures treated with fluorescent nanoparticles. (**A′**–**D′**) Enlarged views corresponding to images in (**A**–**D**) showing colocalization of the DFM signal and the red fluorescence within the cells. (**E**–**H**) Confocal z-projections of OLN-93, N2a, BV-2, and NIH-3T3 cells treated with GNP@PEI-RBITC. PDGFRα (**E**), Tuj-1 (**F**), Iba-1 (**G**), and β-tubulin (**H**) are shown in green, while the red fluorescence corresponds to internalized GNP@PEI-RBITC. Orthogonal views adjacent to each image further confirm the cytoplasmic localization of the nanoparticles. The scale bar in (**A**) represents 15 µm for (**A**–**D**), and the scale bar in (**E**) represents 15 µm for (**E**–**H**).

To further investigate nanoparticle localization, linear PEI was covalently conjugated to rhodamine derivative RBITC, and the resulting labeled polymer was used to synthesize GNP@PEI-RBITC nanoparticles. The amount of fluorophore incorporated per milligram of PEI polymer was quantified (Figure S8) [39]. In treated cell lines, both the scattering signal derived from GNPs and the rhodamine fluorescence were detected by combining dark-field and fluorescence microscopy. However, both signals did not fully overlap. The fact that the rhodamine signal appeared more widespread and diffuse than the gold signal (Figure 7A–D) could reflect differences in the sensitivity of both methods.

The confocal microscopy analysis further confirmed the internalization of GNP@PEI-RBITC, as the fluorescent signal was clearly detected within the cytoplasm of treated cells (Figure 7E–H). Altogether, these results indicate that GNP@PEI was efficiently internalized and distributed within the cytoplasm (Figure S9).

### 3.6. The Intranasal Administration Enables Effective CNS Delivery of GNP@PEI

To assess biodistribution, adult mice received GNP@PEI by either the intranasal (IN) or the intraperitoneal (IP) routes. The gold content in tissues was quantified by inductively coupled plasma tandem mass spectrometry (ICP-MS) after 30 min, 2 h, and 7 d post-administration (*n* = 2 for each time group). No apparent signs of toxicity or behavioral alterations were observed in treated animals throughout the experimental period.

Following the intranasal delivery, the olfactory bulb displayed the highest levels of gold at 30 min (520.5 ± 563.6 ng Au/g tissue). These levels decreased by approximately 50% at 2 h (251.8 ± 240.7 ng/g) and by ten-fold at 7 d (52.6 ± 3.8 ng/g) (Figure 7A). Although values varied among animals, gold levels remained clearly detectable after one week, indicating efficient deposition and retention of GNP@PEI within the olfactory bulb. Gold levels in the brain hemispheres were lower (17.35 ± 1.5 ng/g at 30 min; 13.75 ± 2.3 ng/g at 2 h; 7.10 ± 1.1 ng/g at 7 d) but followed a similar decreasing trend, suggesting a measurable transfer from the olfactory bulb toward deeper brain regions. These findings support the occurrence of direct nose-to-brain transport via olfactory and trigeminal pathways, as previously described for nanoparticle delivery systems [28,29].

**Figure 8:**
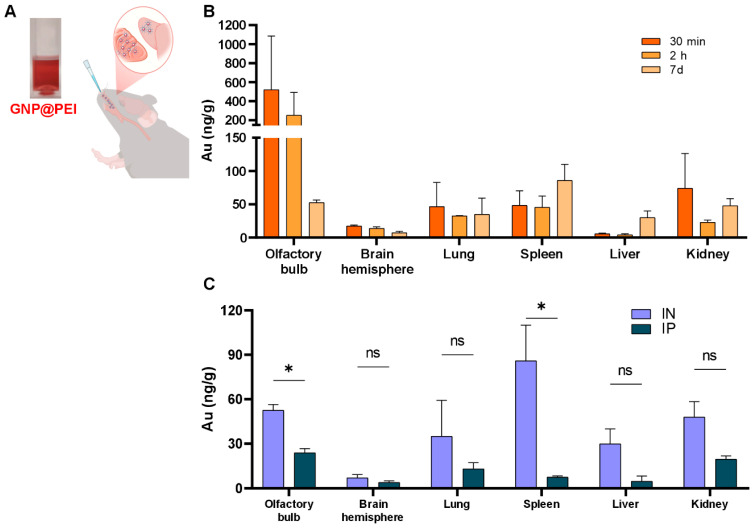
The intranasal administration enables effective CNS delivery of GNP@PEI. (**A**) Schematic representation of the intranasal administration procedure in mice. The illustration depicts the GNP@PEI formulation and its application using a micropipette tip positioned over the mouse nostril. A magnified inset highlights the nose-to-brain transport pathway, illustrating the passage of nanoparticles from the nasal cavity to the olfactory bulb. (**B**) Determination of gold levels (ICP-MS) expressed as ng of Au/g of tissue at different timepoints (30 min, 2 h, and 7 d) following intranasal (IN) delivery of GNP@PEI. Bar graphs display gold accumulation in the analyzed organs, including olfactory bulb, brain hemispheres, lungs, spleen, liver, and kidneys. Bars represent the mean and the standard deviation (SD) (*n* = 2) for each time point. (**C**) Comparison of gold accumulation 7 d after intranasal (IN) vs. intraperitoneal (IP) administration of GNP@PEI, expressed as ng Au/g tissue. Differences between both groups (IN vs. IP) were analyzed by the Student’s *t*-test for each tissue sample. Bars represent the mean and the standard deviation (SD). Asterisks indicate significant differences (* *p* < 0.0332) between IN and IP administration.

Outside the CNS, gold was detected in lung, spleen, liver, and kidney tissues after the intranasal administration (Figure 8A). Gold accumulation in the liver was low at early timepoints (5.9 ± 1.2 ng/g at 30 min) and increased progressively at 7 d (29.9 ± 10.1 ng/g). Similarly, the spleen and kidneys exhibited delayed increases in the Au content (spleen: 48.2 ± 17.6 ng/g at 30 min, 45.5 ± 17.2 ng/g at 2 h, 86.0 ± 17.1 ng/g at 7 d; kidney: 74.3 ± 37.2 ng/g at 30 min, 22.9 ± 3.5 ng/g at 2 h, 48.0 ± 10.4 ng/g at 7 d), consistent with secondary systemic redistribution and reticuloendothelial (RES) uptake of the nanoparticles. Gold was also detected in the lungs (46.6 ± 25.4 ng/g at 30 min, 32.6 ± 0.3 ng/g at 2 h, 34.9 ± 17.9 ng/g at 7 d), likely reflecting partial aspiration of the GNP@PEI formulation. This peripheral distribution pattern is consistent with earlier studies describing progressive hepatic and splenic accumulation of polymer-stabilized gold nanoparticles via RES clearance [23,40,41,42].

When GNP@PEI was administered intraperitoneally, gold was also detected in several organs; however, brain levels were markedly lower than those observed after intranasal administration (Figure 8B). At 7 d, mean Au concentrations (ng/g ± SD) for the IN route vs. IP were as follows: olfactory bulb 52.6 ± 3.8 vs. 23.9 ± 2.8 (ratio IN/IP ≈ 2.2), brain hemispheres 7.1 ± 1.1 vs. 3.9 ± 0.8 (≈1.8), lung 34.9 ± 17.9 vs. 13.1 ± 2.7 (≈2.7), spleen 86.0 ± 17.1 vs. 7.6 ± 0.8 (≈11.4), liver 29.9 ± 10.1 vs. 4.7 ± 1.8 (≈6.4), and kidney 48.0 ± 10.4 vs. 19.6 ± 2.2 (≈2.5). These findings demonstrate that the intranasal delivery provides greater overall tissue exposure and improved CNS bioavailability, as compared to the systemic injection, highlighting the advantage of the IN route in bypassing the blood–brain barrier while maintaining controlled systemic biodistribution [23].

In summary, the intranasal administration of GNP@PEI achieved efficient, sustained, and well-tolerated delivery to the CNS, with measurable gold levels persisting in the olfactory bulb and brain for at least one week after administration. The peripheral accumulation at later timepoints reflects gradual redistribution through the RES, consistent with known biodistribution profiles for gold nanoparticles of similar size (45–55 nm) and positive surface charge (>+30 mV) [42]. Overall, these findings validate the intranasal route as an effective brain-targeted delivery strategy for PEI-stabilized gold nanoparticles, supporting its potential for future neurological and nanotherapeutic applications.

Altogether, these findings demonstrate the biocompatibility, cellular uptake, and effective brain delivery of GNP@PEI, providing a comprehensive characterization of their behavior in vitro and in vivo.

## 4. Discussion

The development of novel therapeutic strategies targeting the central CNS continues to face numerous challenges, including the extensive heterogeneity of nervous tissues and the restricted permeability imposed by the blood-brain barrier, which limits drug access into the brain parenchyma. Nanoparticles have emerged as promising platforms to overcome these limitations due to their tunable physicochemical properties, capacity to cross biological barriers, and capacity to carry or allow conjugation to bioactive molecules [43,44,45,46,47]. In this context, our results show that gold nanoparticles (GNPs) coated with 22 kDa linear PEI exhibited high biocompatibility in both neural and non-neural cell types, including primary cultures of neural stem cells (NSCs) and microglia cells, as well as OLN93, BV2, N2A, and NIH3T3 cell lines. These findings are consistent with previous studies reporting that GNPs generally have low cytotoxicity and are well-tolerated in vitro and in vivo, particularly when coated with biocompatible polymers [48,49]. The fact that the treatment with GNP@PEI did not affect the viability of several cell types makes this approach a suitable candidate for CNS-targeted delivery. Among the tested cell types, NSCs showed the greatest sensitivity, with a mild decrease in (metabolic activity) viability at 10 ppm GNP@PEI, a phenomenon that may relate to the high proliferative rate or metabolic demands of this cell type. While the current study focused on cell viability, we also noted a decrease in cell proliferation in response to the treatment. The mechanism behind this effect is the subject of future research, as understanding this pathway could provide novel insights into the effects of GNP@PEI on NSC biology and the treatment’s full potential.

The selection of linear 22 kDa PEI·HCl as the coating polymer was based on preliminary studies in which NSC cultures were exposed to PEI of different molecular weights. In these experiments, the PEI·HCl polymer exhibited lower toxicity on NSCs compared with PEI·HCl polymers of higher molecular weight. This is consistent with previous studies showing that the molecular weight critically influences the biological behavior of PEI, with PEI·HCl of molecular weights higher than 22 kDa exhibiting markedly higher toxicity compared with lower molecular weight variants [13,50]. However, the optimal length of a polymer conjugated to nanoparticles depends on the specific application and desired properties of the resulting nanohybrid. Our results showed that the PEI·HCl (22 kDa) served as a reducing agent while enabling efficient nanoparticle formation and stabilization. Under the reaction conditions used for the synthesis, we obtained homogeneous PEI-coated nanoparticles with a mean diameter of ~50 nm, which, as an additional advantage, remained highly stable in water for at least six months. Both the coordination of AuCl_4_^−^ ions and the oxidation of PEI secondary amine groups to their corresponding imine moieties have been directly monitored and demonstrated by ^1^H-NMR experiments in D_2_O. Additionally, this eco-friendly synthetic approach eliminates the need for toxic reductants, is in compliance with green chemistry principles, and yields highly stable positive nanosystems, as confirmed by DLS, XPS, and HR-TEM analyses.

Dark field microscopy of primary cell cultures treated with GNP@PEI revealed the presence of nanoparticles inside both proliferative and non-proliferative cells, suggesting that internalization is not restricted to a specific phase of the cell cycle. Similar findings were obtained in all tested cell lines. However, since dark-field microscopy cannot distinguish between membrane-bound and internalized particles, we employed rhodamine-labeled GNP@PEI to examine the intracellular particle distribution. The confocal microscopy analysis demonstrated a cytoplasmic localization of GNP@PEI-RBITC, indicating that these nanoparticles were effectively taken up by the cells and accumulated within the intracellular compartment. Nevertheless, the gold-associated scattering signal and the rhodamine fluorescence did not fully overlap. Similar discrepancies between plasmonic scattering and fluorescence signals have been previously reported for other dye-functionalized GNPs. These differences arise because each method detects different physical phenomena (scattering vs. emission), and the resulting signals do not necessarily colocalize. For instance, particles easily detectable by dark-field microscopy may not be visible by fluorescence microscopy due to factors such as fluorophore photobleaching or low fluorescence sensitivity [51]. Consistent with these observations, fluorescence microscopy provides a convenient and powerful imaging method, whereas DFM offers a complementary approach with superior sensitivity for detecting nanoparticle-associated scattering signals. While we confirmed the presence of nanoparticles inside the cells, future studies employing electron microscopy are required to define subcellular localization, endocytic pathways, and fate within the endo-lysosomal system. While dark-field and confocal microscopies provided qualitative and spatial evidence of intracellular localization, the present study did not quantify the absolute intracellular gold mass (e.g., by ICP-MS of washed cell pellets/lysates). This constitutes a limitation and will be addressed in future works.

Importantly, in vivo experiments confirmed that intranasally administered GNP@PEI reached the CNS, with gold accumulation detected in the olfactory bulb and brain hemispheres as early as 30 min post-administration. The intranasal route relies on the anatomical connection between the nasal mucosa and the CNS, allowing nanoparticles to bypass the blood-brain barrier via the olfactory and trigeminal nerve pathways [52]. This strategy has previously been validated in studies using various nanocarriers, including polymeric NPs and liposomes [22,53]. Notably, we observed a progressive decline in brain gold levels over one week, with a corresponding increase in the liver and consistent with a systemic redistribution and clearance through detoxification organs [42]. Gold was detected in peripheral organs, including the lung and liver, consistent with partial aspiration following intranasal dosing (lung) and reticuloendothelial redistribution/clearance (liver and spleen). In this proof-of-concept study, animals showed no overt signs of distress and no more than 5% body weight loss during the observation period; however, dedicated organ-toxicity endpoints (histopathology, serum biochemistry, and inflammatory markers) were not assessed and should be included in future work to comprehensively evaluate organ-specific safety. A further limitation of the in vivo biodistribution component is the inclusion of male mice only; planned studies will incorporate both sexes to better capture biological variability. From a translational perspective, the combination of biocompatibility, aqueous stability, and the capacity of GNP@PEI to reach neural tissues positions this nanoplatform as a promising tool for CNS-targeted delivery with potential applications in both neurology and neuro-oncology. Moreover, our results suggest that this strategy could be used to functionalize nanoparticles with therapeutic cargos such as siRNA, anti-inflammatory agents, or pro-oligodendrogenic factors, which may be used to modulate the neural stem cell fate or to promote remyelination in demyelinating diseases, as previously proposed [24,54,55]. It is also important to highlight that several gold-based nanoplatforms have advanced to phase II/III clinical trials for the treatment of neurodegenerative diseases [6,56], underscoring the rapid growth of this field and the significant potential of nanomedicine for addressing disorders of the nervous system. However, some limitations of this platform must be considered. The long-term safety and immunogenicity of these systems require thorough investigation to assess the risks associated with chronic exposure, especially considering that positively charged nanoparticles have been associated with pro-inflammatory responses in some settings [57]. In addition, particle size, charge, and surface chemistry strongly influence brain penetration and cell-type specificity; therefore, future work should systematically explore these parameters to optimize CNS delivery. Notably, the simplicity and green-chemistry profile of the synthetic method employed in this work further increase the feasibility of scaling up the GNP@PEI production toward preclinical development.

In summary, our findings highlight the potential of linear PEI-coated GNPs as a robust, biocompatible, versatile platform for CNS drug delivery. Their high stability in water, efficient nose-to-brain transport, low toxicity in several neural and non-neural cell types, and the capacity to interact with key neural cell populations make them useful tools for the development of novel treatments for neurological disorders.

## 5. Conclusions

In this study, we successfully synthesized and characterized GNP@PEI, demonstrating their high biocompatibility in both neural and non-neural cell types, as well as efficient internalization by primary neural stem cells, microglia, and various brain-derived cell lines.

The GNP@PEI was studied using a wide range of spectroscopic techniques (UV-Visible, HR-TEM, DLS, FT-IR, XPS, and NMR), among which it is noteworthy that it was possible to follow the chemical changes in the PEI·HCl molecules during the formation of the GNPs through ^1^H-NMR experiments in D_2_O.

Importantly, in vivo biodistribution studies confirmed that GNP@PEI can bypass the blood-brain barrier through the nose-to-brain route upon intranasal administration, offering a minimally invasive and efficient strategy to deliver nanocarriers to the CNS. The observed progressive redistribution of gold to detoxifying organs suggests the activation of physiological clearance pathways, supporting the biosafety of these nanoparticles over time.

In summary, we established a reproducible one-step bottom-up synthesis of GNP@PEI with long-term colloidal stability and high positive surface charge. We demonstrated broad biocompatibility and cell-type-dependent uptake across primary NSCs/microglia and multiple CNS-relevant cell lines. Importantly, ICP-MS biodistribution revealed measurable gold in brain regions following intranasal delivery, with persistence up to 7 d, and a distinct distribution profile compared to intraperitoneal administration. Taken together, our findings support the use of GNP@PEI as a versatile platform for CNS-targeted drug delivery. Future studies should evaluate both long-term safety and therapeutic performance, focusing on the functionalization of these nanocarriers with therapeutic molecules to modulate neural stem cell behavior or to interfere with pathological mechanisms involved in neurological diseases, including neurodegeneration, gliomas, and demyelinating disorders. Altogether, these properties position GNP@PEI as a promising nanoplatform with clear potential for translation into future CNS therapies and motivate future studies incorporating uptake quantification and expanded safety endpoints.

## Figures and Tables

**Table 1:** Hydrodynamic diameter and PDI values of GNP@PEI in deionized water at 25 °C monitored over a six-month period to assess long-term stability of the colloidal dispersion at 4 °C.

Sample	Hydrodynamic Diameter by Intensity (nm) (±SD)				PDI (±SD)
	Peak 1	%	Peak 2	%	
GNP@PEI 1st Month	46.9 (0.2)	94.4 (0.2)	5.1 (0.3)	5.5 (0.2)	0.267 (0.004)
GNP@PEI 2nd Month	45.3 (0.9)	93.9 (0.5)	5.2 (0.4)	6.1 (0.5)	0.262 (0.010)
GNP@PEI 3rd Month	46.4 (0.6)	93.8 (0.4)	5.2 (0.2)	6.2 (0.4)	0.263 (0.002)
GNP@PEI 4th Month	42.4 (0.8)	91.3 (0.4)	3.4 (0.2)	8.7 (0.4)	0.322 (0.004)
GNP@PEI 5th Month	41.3 (2.1)	90.6 (0.8)	3.7 (0.2)	9.0 (0.6)	0.349 (0.048)
GNP@PEI 6th Month	41.3 (0.8)	90.8 (0.1)	3.6 (0.1)	9.2 (0.1)	0.358 (0.059)

## Data Availability

The original contributions presented in this study are included in the article/Supplementary Materials. Further inquiries can be directed to the corresponding authors.

## Supplementary Materials

The following supporting information can be downloaded at: https://www.mdpi.com/article/10.3390/polym18020298/s1, Figure S1: Effects of linear PEI·HCl polymers on the metabolic activity of neural stem cells; Figure S2: Characterization of GNP@PEI synthesized with PEI of different molecular weights and gold ratios; Figure S3: Long-term colloidal stability of GNP@PEI; Figure S4: Effect of lyophilization on the structural integrity of GNP@PEI; Figure S5: Elemental mapping of GNP@PEI by STEM–EDX; Figure S6: High resolution (A) Au 4*f*, (B) C 1*s* and (C) N 1*s* core level spectra for the GNP@PEI sample; Figure S7: ATR-FTIR spectra of PEI and GNP@PEI; Figure S8: Spectrophotometric quantification of rhodamine (RBITC) labeling; Figure S9: Confocal microscopy stock images for the OLN-93, N2a, BV-2 and NIH-3T3 cells treated with GNP@PEI-RBITC. Tables S1: Fitting parameters from N 1*s*, Au 4*f* and C 1*s* XPS data of the GNP@PEI sample. Video S1: Visual record of GNP@PEI synthesis; Video S2: Phagocytosis of GNP@PEI by primary rat microglia; ARRIVE Checklist.
