# Supplementary material for: Linear Polyethyleneimine-Coated Gold Nanoparticles as a Platform for Central Nervous System Targeting
Source: Polymers (Basel). 2026 Jan 22;18(2):298. doi: 10.3390/polym18020298 (PMC12845547; doi:10.3390/polym18020298)
Supplement: Supplementary file 1 [file polymers-18-00298-s001.zip › Supplementary Material.pdf]

"Supplementary Material"

Linear polyethyleneimine-coated gold nanoparticles as a platform for central nervous system targeting

Agustín J. Byrne, Antonia Infantes-Molina, Enrique Rodríguez-Castellón, Romina J. Glisoni, María J. Pérez, Patrizia Andreozzi, Barbara Richichi, Marco Marradi, Paula G. Franco* and Juan M. Lázaro-Martínez*

Table of Contents:

Content	***Page***
Figure S1	2
Figure S2	3
Figure S3	4
Figure S4	5
Figure S5	6
Figure S6	7
Table S1	7
Figure S7	8
Figure S8	9
Figure S9	9
Video S1 (Caption)	9
Video S2 (Caption)	9

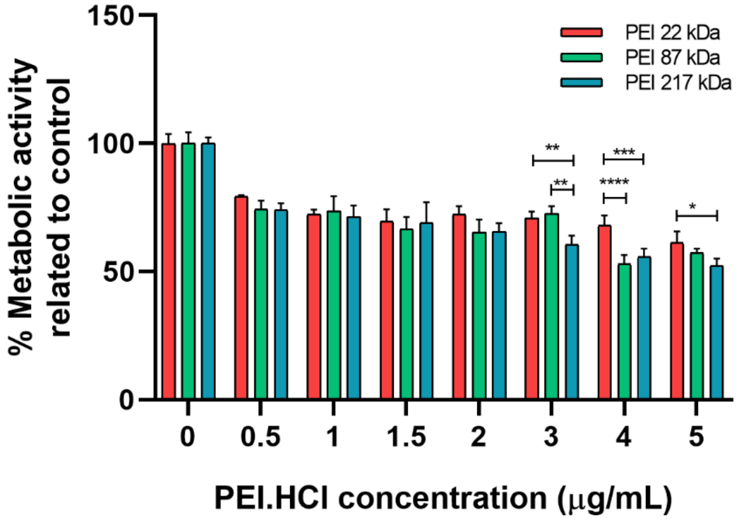

Figure S1. Effects of linear PEI·HCl polymers on the metabolic activity of neural stem cells. Percentage of metabolic activity relative to untreated control determined by MTT assay in primary neural stem cell cultures exposed to different molecular weights of linear PEI·HCl. All tested concentrations showed statistically significant differences compared to their respective controls. Data are presented as mean ± SD. ****$p < 0.0001$, ***$p < 0.001$, **$p < 0.01$, *$p < 0.05$ *vs.* indicated groups.

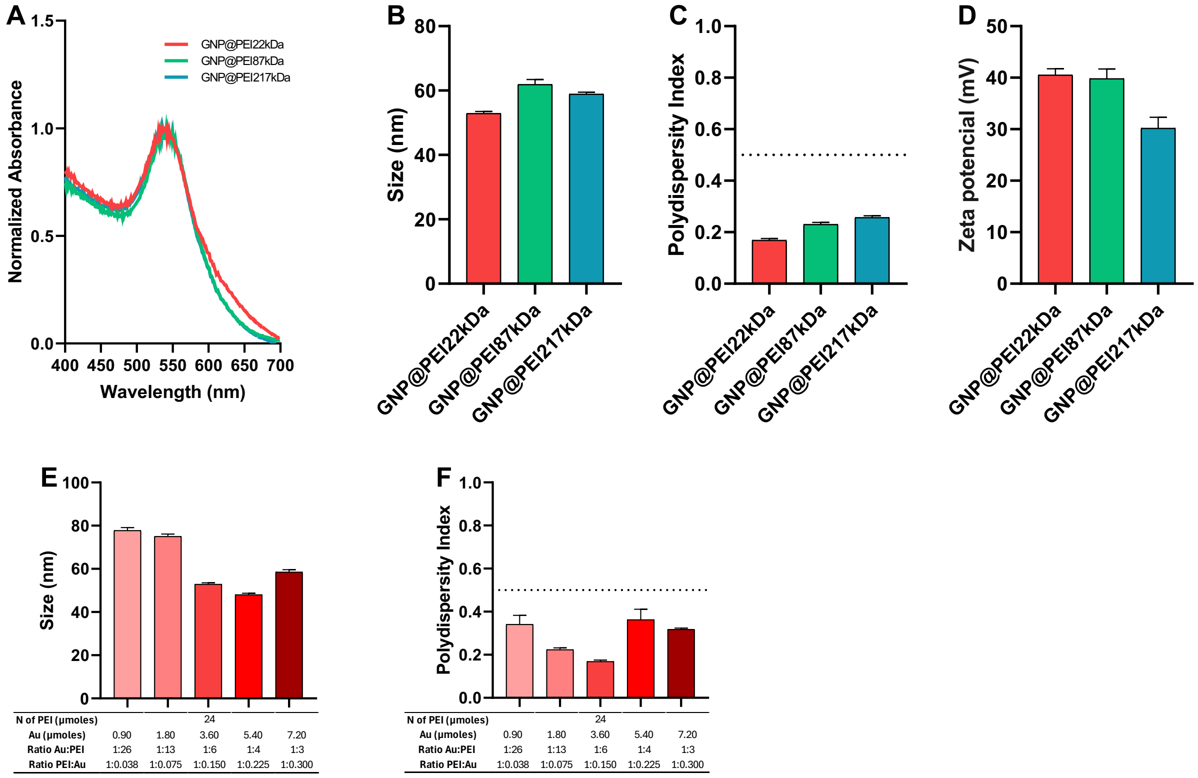

Figure S2. Characterization of GNP@PEI synthesized with PEI of different molecular weights and gold ratios. (A) UV–Vis absorption spectra of GNP@PEI synthesized using linear PEI of 22, 87, and 217 kDa, showing the characteristic surface plasmon resonance band of gold nanoparticles. (B–D) Colloidal parameters of GNP@PEI measured by dynamic light scattering (DLS) in deionized water at 25 °C: (B) hydrodynamic diameter, (C) polydispersity index (PDI), and (D) zeta potential. (E, F) Hydrodynamic diameter (E) and corresponding PDI values (F) of GNP@PEI synthesized with 22 kDa PEI under varying $HAuCl_4$ concentrations, measured by DLS in deionized water at 25 °C.

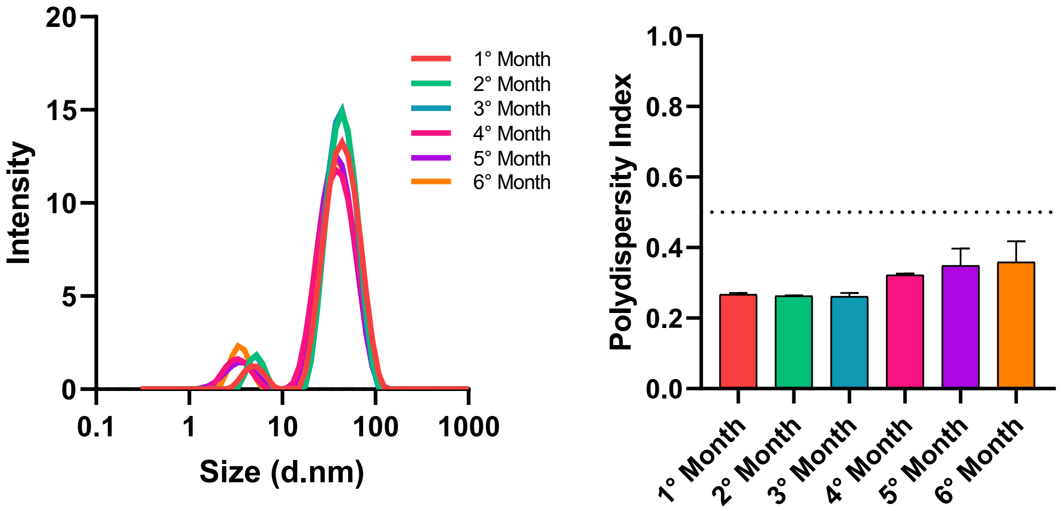

Figure S3. Long-term colloidal stability of GNP@PEI. (A) Hydrodynamic size distribution by intensity (%) measured by DLS over a six-month storage period at 4°C. (B) Corresponding polydispersity index (PDI) values represented as bar plots, demonstrating the long-term stability of the colloidal suspension at 4°C.

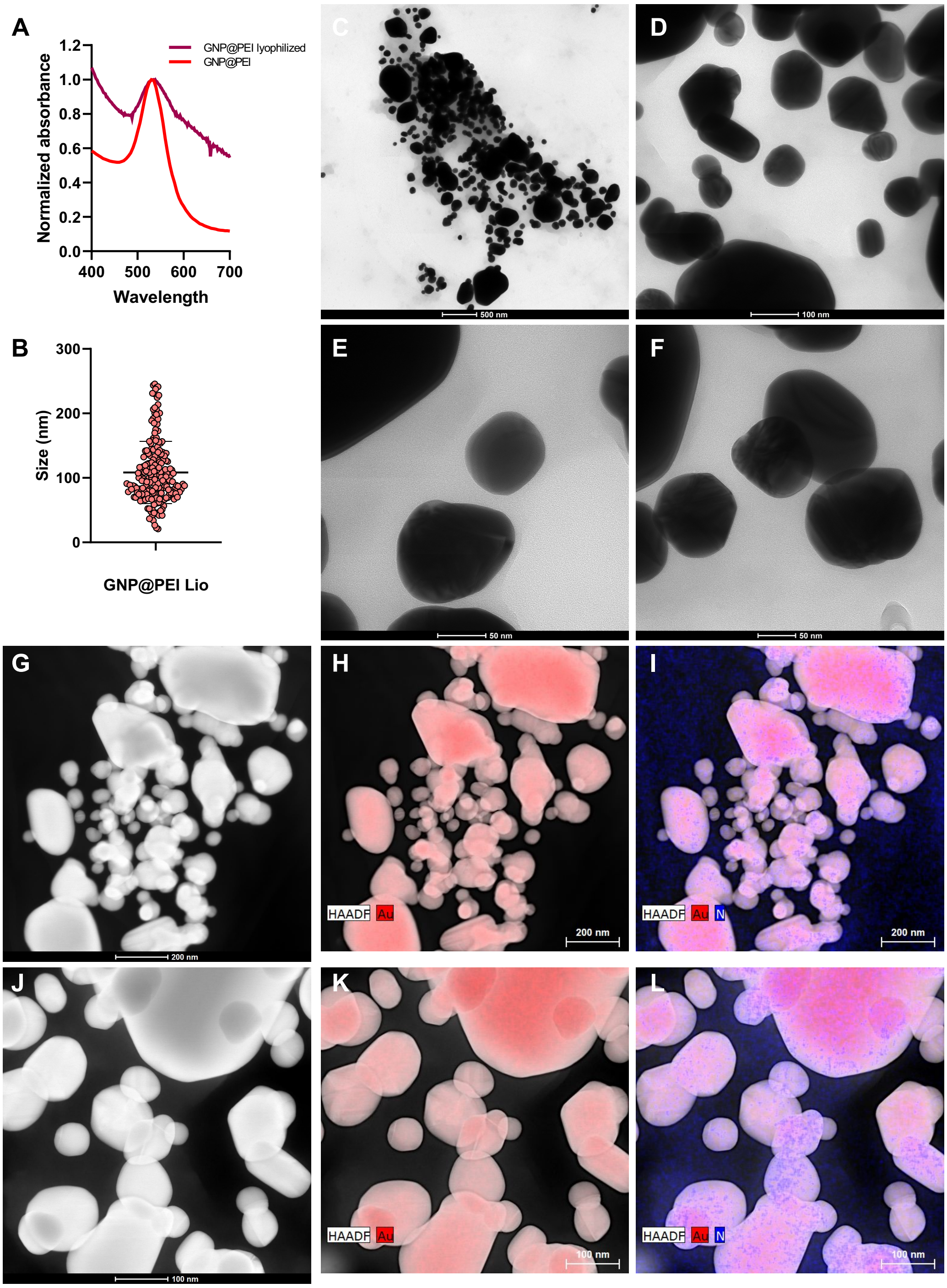

Figure S4. Effect of lyophilization on the structural integrity of GNP@PEI. *(A)* UV–Vis spectra of freshly prepared (red line) and lyophilized GNP@PEI (purple line) normalized to A/A_{max}. The lyophilized sample exhibited a broadening and red-shift of the surface plasmon resonance (SPR) band, indicative of particle aggregation. *(B)* Quantitative analysis of HRTEM micrographs showing an average particle size of ~108 nm after lyophilization, compared to ~47 nm in non-lyophilized samples. *(C–F)* Representative HRTEM images of lyophilized GNP@PEI at different magnifications, revealing extensive nanoparticle aggregation and a few isolated particles, particularly in panels (E) and (F). Panels (G–L) correspond to STEM–EDX analyses. *(G and J)* HAADF-STEM images of GNP@PEI aggregates at different magnifications, and the corresponding elemental mappings for Au (H, K) and N (I, L), confirming the distribution of gold-rich regions surrounded by nitrogen-containing polymeric material. All images include scale bars. Panels (G–L) correspond to STEM–EDX analyses.

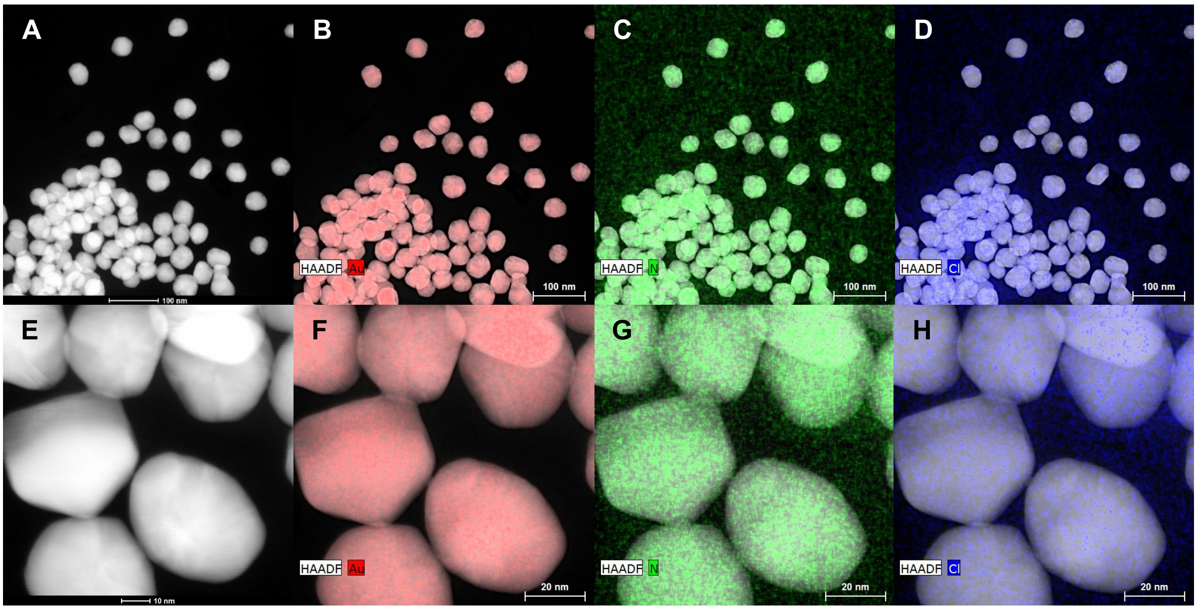

Figure S5. Elemental mapping of GNP@PEI by STEM–EDX. Representative STEM–EDX images showing nanoparticle morphology and elemental composition. (A, E) High-Angle Annular Dark-Field Scanning Transmission Electron Microscopy (HAADF) images; (B, F) gold (Au) distribution; (C, G) nitrogen (N) distribution; and (D, H) chlorine (Cl) distribution, confirming the presence of PEI and residual chloride species on the nanoparticle surface.

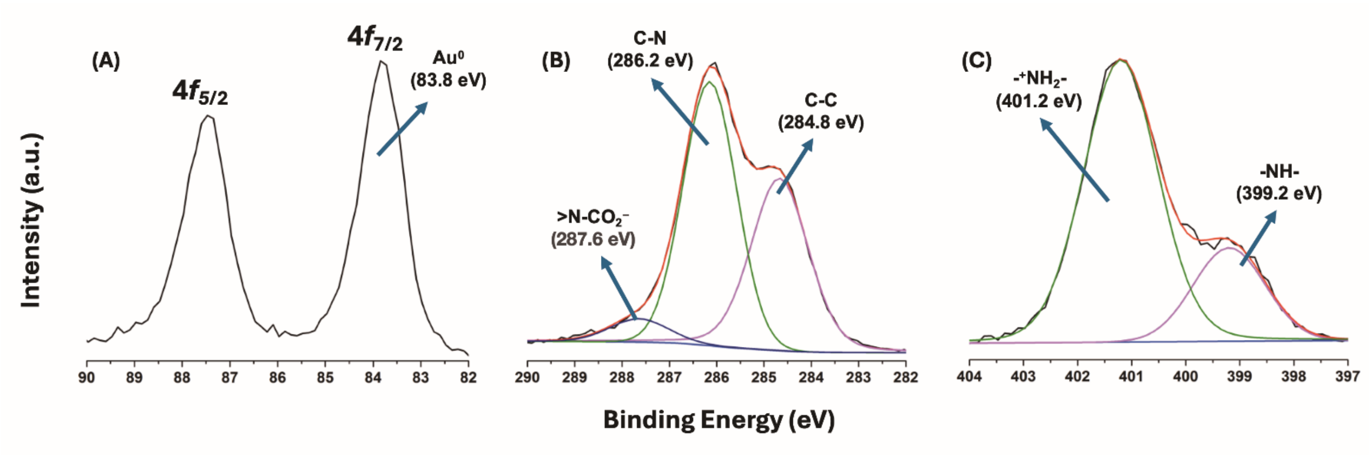

Figure S6. High resolution (A) Au 4*f*, (B) C 1*s* and (C) N 1*s* core level spectra for the GNP@PEI sample.

Table S1. Fitting parameters from N 1*s*, Au 4*f* and C 1*s* XPS data of the GNP@PEI sample.

**N 1*s***								
**Band**	**Pos**	**PosSep**	**B_FWHM**	**FWHM**	**Height**	**%Gauss**	**Area**	**%Area**
**1**	**399.2**	**0**	**1.56**	**1.56**	**1017**	**100**	**1691**	**23.18**
**2**	**401.2**	**2**	**1.62**	**1.62**	**3071**	**88**	**5603**	**76.82**
**Au 4*f***								
**Band**	**Pos**	**PosSep**	**B_FWHM**	**FWHM**	**Height**	**%Gauss**	**Area**	**%Area**
**1**	**83.8**	**0**	**1.1**	**1.1**	**408**	**80**	**523**	**57.14**
**2**	**87.5**	**3.67**	**1.1**	**1.1**	**310**	**83**	**392**	**42.86**
**C 1*s***								
**Band**	**Pos**	**PosSep**	**B_FWHM**	**FWHM**	**Height**	**%Gauss**	**Area**	**%Area**
**1**	**284.8**	**0**	**1.42**	**1.42**	**3922**	**80**	**6515**	**41.02**
**2**	**286.2**	**1.48**	**1.33**	**1.33**	**6009**	**100**	**8510**	**53.58**
**3**	**287.6**	**2.97**	**1.46**	**1.46**	**529**	**90**	**859**	**5.41**

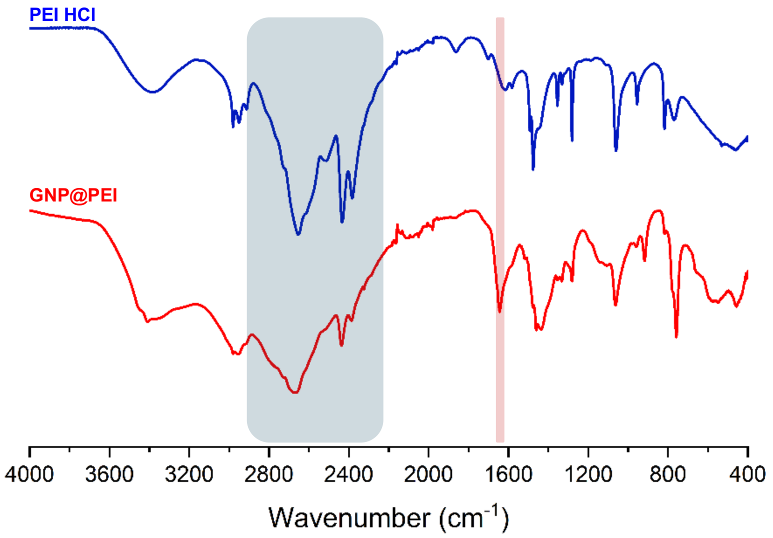

Figure S7. ATR-FTIR spectra of PEI and GNP@PEI. ATR-FTIR spectra showing characteristic polymer signals retained in GNP@PEI. The band at 1646 cm^{-1} in GNP@PEI corresponds to C = N stretching (red-shaded area), consistent with imine formation during PEI oxidation. Additional changes are observed in the 2900–2300 cm^{-1} region (blue-shaded area), associated with the strong intermolecular hydrogen-bonding network characteristic of PEI.

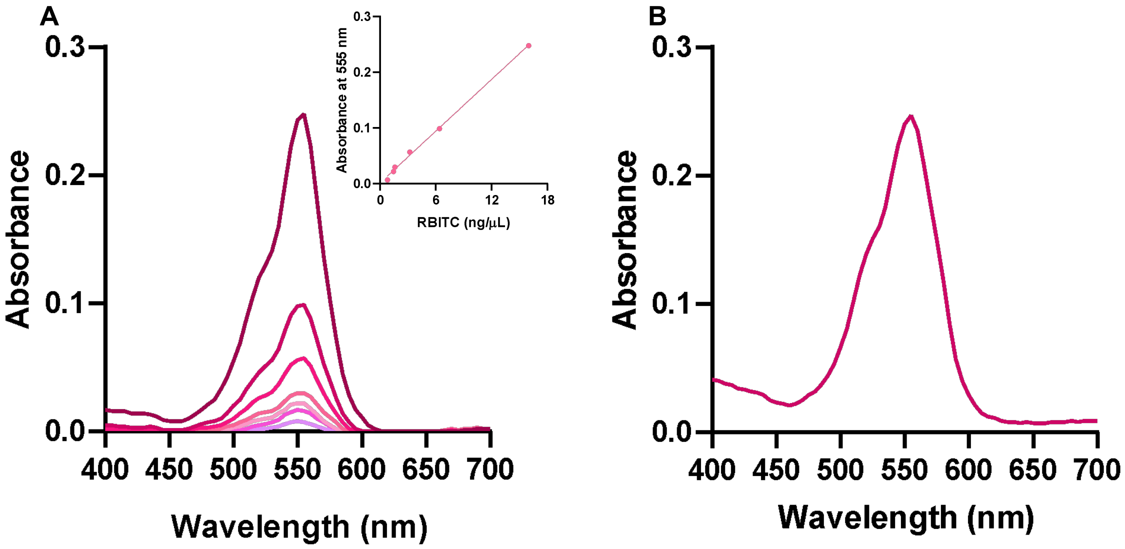

Figure S8. Spectrophotometric quantification of rhodamine (RBITC) labeling. (A) UV–Vis spectrum of rhodamine (RBITC) and calibration curve obtained by plotting absorbance at 555 nm *vs.* concentration (ng/µL). (B) Absorption spectrum of PEI–Rho conjugate used to estimate the amount of bound rhodamine per mg of PEI based on the calibration curve.

Figure S9. Confocal microscopy stock images for the OLN-93, N2a, BV-2 and NIH-3T3 cells treated with GNP@PEI-RBITC. (Check "Figure S9.zip" file).

Video S1. Visual record of GNP@PEI synthesis. Time-lapse showing the color change during GNP@PEI synthesis, from bright yellow (PEI–Au complex) to deep carmine red (colloidal GNP formation). The timer displayed in the video corresponds to a real-time scale where each second equals one minute of reaction. (Check "Video S1.mp4" file).

Video S2. Phagocytosis of GNP@PEI by primary rat microglia. Primary microglial cultures exposed to GNP@PEI showing the active uptake of gold nanoparticles. Bright, highly refractive spots corresponding to GNPs can be observed within the cytoplasm, moving dynamically as the particles are internalized and redistributed inside the cells. Images were recorded in real time under dark-field microscopy. (Check "Video S2.mp4" file).
